# Diversity and Fungicide Sensitivity of *Colletotrichum* spp. Causing Apple Bitter Rot and Glomerella Leaf Spot in Northwestern Italy

**DOI:** 10.3390/plants15203121

**Published:** 2026-10-12

**Authors:** Ilaria Martino, Eleonora Raschio, Greta Dardani, Giuseppe Massimo Pichierri, Davide Spadaro, Vladimiro Guarnaccia

**Affiliations:** 1 Department of Agricultural, Forest and Food Sciences (DISAFA), University of Torino, Largo P. Braccini 2, 10095 Grugliasco, TO, Italy; eleonora.raschio@unito.it (E.R.); greta.dardani@unito.it (G.D.); giuseppemassimo.pichierri@unito.it (G.M.P.); davide.spadaro@unito.it (D.S.); vladimiro.guarnaccia@unito.it (V.G.); 2 AGROINNOVA — Interdepartmental Centre for Innovation in the Agro-Environmental Sector, University of Torino, Largo P. Braccini 2, 10095 Grugliasco, TO, Italy

**Keywords:** *Malus* × *domestica*, *Colletotrichum chrysophilum*, fludioxonil, mefentrifluconazole, pyraclostrobin

## Abstract

Apple bitter rot (ABR) and Glomerella leaf spot (GLS), caused by *Colletotrichum* spp., have recently become an increasing concern in most Italian apple-growing areas. Surveys were conducted between 2023 and 2025 in 28 commercial apple orchards in Piedmont, Northwestern Italy, to investigate the occurrence and diversity of *Colletotrichum* species associated with symptomatic fruits and leaves. A total of 155 isolates were obtained and initially screened using *gapdh*, while 49 representative isolates were selected for species-level identification through multilocus phylogenetic analyses. Five species belonging to the *C. gloeosporioides* and *C. acutatum* species complexes were identified: *C. chrysophilum*, *C. alienum*, *C. fioriniae*, *C. godetiae*, and *C. nymphaeae*. Eight representative isolates were evaluated for mycelial growth at different temperatures and pathogenicity on apple fruits and leaves, revealing differences in growth and virulence. All tested isolates were pathogenic and fulfilled Koch’s postulates. Isolates were characterized for their in vitro sensitivity to fludioxonil, mefentrifluconazole, and pyraclostrobin. EC_50_ values were generally low, although greater variability was observed, particularly within the *C. gloeosporioides* species complex for pyraclostrobin. This study expands current knowledge of the *Colletotrichum* species associated with ABR and GLS in apple production and provides relevant biological and fungicide sensitivity data as a reference for future epidemiological investigations and disease management strategies.

## 1. Introduction

Apple (*Malus* × *domestica*) is one of the world most valuable temperate fruit crops, with global production reaching approximately 97.9 million metric tons in 2024, led by China (51.3 million tons), followed by the USA (4.9 million tons), Türkiye (4.4 million tons), Poland (3.4 million tons), and India (2.6 million tons) [1]. Italy ranks sixth within the global production, with 55,030 cultivated hectares and over 2.3 million tons produced in 2025, mainly concentrated in Northern regions, including Trentino Alto Adige, Veneto, Emilia Romagna, and Piedmont [2].

Several fungal diseases are infecting apple leaves and fruits, both in pre- and post-harvest conditions, across Italy, particularly in Northern production areas, causing considerable yield and quality losses. Apple scab caused by *Venturia inaequalis* is the most destructive disease affecting apple in this area followed by powdery mildew caused by *Podosphaera leucotricha* [3,4]. Postharvest diseases are mainly caused by wound-infecting fungi including *Penicillium*, *Botrytis*, *Rhizopus*, and *Mucor* species [5]. Latent pathogens such as *Neofabraea* spp., causing bull’s eye rot, and *Ramularia mali*, causing dry lenticel rot, as well as white haze disorder, associated with different basidiomycete genera, can further compromise fruit quality and marketability [6,7,8,9].

Since 2021–2022, outbreaks of Glomerella leaf spot (GLS) and apple bitter rot (ABR) have been reported with high incidence across Northern Italian apple orchards [10,11,12,13]. GLS is characterized by small spots that enlarge into necrotic lesions on leaves, often leading to chlorosis and premature defoliation; acervuli and, in some cases, perithecia may develop on the leaf surface [14,15]. In contrast, typical ABR symptoms consist of light-brown circular lesions on fruits that progressively become sunken and dark, frequently showing concentric circles of acervuli from which salmon-orange conidial masses emerge [15,16]. More than 20 *Colletotrichum* species mainly belonging to the *C. acutatum* and *C. gloeosporioides* species complexes (SCs) have been associated with ABR and GLS worldwide [14]. Historically, *Colletotrichum* spp. in Italy were mainly associated with latent infections and postharvest storage rots of apple fruits [17,18]. However, their recent and consistent isolation from symptomatic leaves and fruits under preharvest conditions represents an emerging threat and raises serious concerns for growers. Previous surveys conducted in Northern Italy identified multiple species associated with GLS and ABR, such as *C. alienum*, *C. chrysophilum*, *C. fioriniae*, *C. grossum*, *C. nymphaeae*, *C. siamense*, and *C. sojae* [11,12,19]. In particular, the increasing detection of species belonging to the *C. gloeosporioides* SC, previously considered uncommon in European apple orchards, suggests a change in *Colletotrichum* spp. composition associated with these diseases and raises questions about their epidemiology. The high diversity of *Colletotrichum* species associated with apple may complicate disease management, as both inter- and intraspecific variations in virulence, growth responses to temperature, and fungicide sensitivity have been reported [14,16].

Italian commercial apple orchards are routinely treated throughout the growing season with fungicides targeting other major pathogens, particularly *Venturia inaequalis* and *Podosphaera leucotricha*. Spray programs include active ingredients belonging to different FRAC groups, such as demethylation inhibitors (DMIs; e.g., mefentrifluconazole and difenoconazole), quinone outside inhibitors (QoIs; e.g., pyraclostrobin and trifloxystrobin), succinate dehydrogenase inhibitors (SDHIs; e.g., fluxapyroxad and boscalid), and multi-site fungicides (e.g., captan, sulfur, and dithianon), which are extensively applied throughout the growing season for the management of apple scab and powdery mildew [20]. Consequently, *Colletotrichum* populations associated with apple are likely repeatedly exposed to several fungicides. Recent studies on apple-associated *Colletotrichum* spp. reported differences in fungicide sensitivity at species and population level, highlighting the importance of monitoring fungicide sensitivity in local populations [21,22,23,24,25,26]. Recently, fludioxonil- and dithianon-based products received label extensions in Italy for the management of *Glomerella*-associated diseases on apple [27]. Nevertheless, information regarding the fungicide sensitivity of Italian *Colletotrichum* isolates associated with apple remains extremely limited, despite the increasing incidence of GLS and ABR in commercial orchards. Accurate species identification together with the evaluation of pathogenicity, virulence, temperature-dependent growth, and fungicide sensitivity is thus essential to improve the understanding of the etiology and epidemiology of *Colletotrichum*-associated diseases and to support the development of sustainable management strategies.

Although the occurrence of several *Colletotrichum* species associated with GLS and ABR has already been documented in Northern Italy, an integrated characterization of their biological traits, pathogenicity, and fungicide sensitivity in Italian apple orchards is still lacking. Therefore, the present study therefore aimed to: (i) identify the main *Colletotrichum* species in association with ABR and GLS in Northwestern Italy using molecular and phylogenetic analyses, (ii) determine the mycelial growth rate of the identified species at different temperatures, (iii) test their pathogenicity to fulfil Koch’s postulates, and (iv) assess their in vitro sensitivity to fludioxonil (phenylpyrrole, FRAC 12), mefentrifluconazole (demethylation inhibitor—DMI, FRAC 3), and pyraclostrobin (quinone outside inhibitor—QoI, FRAC 11).

## 2. Results

### 2.1. Field Sampling and Fungal Isolation

Symptoms consistent with GLS and ABR were observed in the surveyed orchards during the study (Table S1). GLS symptoms consisted of small necrotic lesions, frequently surrounded by chlorotic halos, that progressively enlarged and coalesced, resulting in extensive leaf blighting (Figure 1). Symptoms were predominantly observed on leaves in the basal part of the canopy and, in severe cases, were associated with extensive early defoliation (Figure 1a,b). Individual lesions appeared as reddish to brown necrotic spots, occasionally surrounded by chlorotic tissue (Figure 1c,d).

ABR symptoms (Figure 2) initially appeared as small spots that progressively enlarged into circular, sunken lesions. As disease progressed, lesions became darker and frequently developed acervuli producing salmon-colored conidial masses (Figure 2c,d).

A collection of 155 *Colletotrichum* isolates was obtained from symptomatic leaves and fruits. Among the collected isolates, 80 originated from leaves and 75 from fruits.

### 2.2. Phylogenetic Analyses

Multilocus phylogenetic analyses were performed separately for the *C. gloeosporioides* and *C. acutatum* SCs using reference strains, including ex-type and epitype strains whenever available (Table S2). The concatenated *gapdh*, *chs-1*, *act*, *tub2*, *cal*, *gs*, and ApMat dataset of the *C. gloeosporioides* SC consisted of 120 strains (83 reference strains retrieved from GenBank and 37 isolates from the present collection) and 3570 characters (*gapdh*: 1–201, *chs-1*: 208–436, *act*: 443–661, *tub2*: 668–1246, *cal*: 1253–1957, *gs*: 1964–2756 and ApMat: 2763–3570). Detailed characteristics of the phylogenetic analyses, including alignment statistics, parsimony parameters, and evolutionary models, are reported in Tables S3 and S4. Because MP and BI analyses produced congruent topologies, only the BI phylogram is shown in Figure 3, with MP bootstrap values reported at the nodes. A total of 36 isolates clustered together with the ex-type strains of *C. chrysophilum* (CMM4268) and 2 additional reference strains of *C. chrysophilum* (CPO 27.222 and LM1270) whereas a single isolate grouped with *C. alienum* type strain (ICMP 12071) and one other *C. alienum* reference strains (CBS 133930) as previously reported [19].

The combined *gapdh*, *chs-1*, *his3*, *act*, and *tub2* dataset of the *C. acutatum* SC comprised 68 strains (55 reference strains retrieved from GenBank, and 12 isolates from the present collection) and 1629 characters (*gapdh*: 1–266, *chs-1*: 273–548, *his3*: 555–902, *act*: 909–1126, *tub2*: 1133–1629). The BI consensus tree, including MP bootstrap support values, is presented in Figure 4.

Alignment statistics, phylogenetic parameters, and substitution models for each locus are summarized in Tables S3 and S4. The 12 isolates analyzed were assigned to three species. Four isolates, one already reported by Martino et al. [19], clustered with ex-type strain of *C. nymphaeae* (CBS 515.78), two additional reference strains of this species (CBS 129945 and IMI 370491) and the ex-type of *C. simulanticitri* (YMF1.07302) currently regarded as a synonym of *C. nymphaeae* [28]. Six isolates grouped with *C. fioriniae* ex-type strain (CBS 128517) and the ex-type strains of *C. orientale* (CBS 128555) and *C. radermacherae* (GZAAS 21-0300), recently synonymized with *C. fioriniae* [29]. Two isolates clustered with *C. godetiae* ex-type strain (CBS 133.44) and two additional reference strains (CBS 127561 and IMI 351248). Sequences generated in this study were deposited in GenBank and their accession numbers are reported in Table 1, together with the sequences of isolates CVG 2563 and CVG 2470 [19].

### 2.3. Effect of Temperature on Mycelial Growth

Eight representative isolates were selected for subsequent biological characterization: CVG 2240 and CVG 2255 (*C. chrysophilum*), CVG 2457 and CVG 2517 (*C. fioriniae*), CVG 2466 (*C. godetiae*), CVG 2470 and CCI 208 (*C. nymphaeae*), and CVG 2563 (*C. alienum*). Isolates differed significantly in both optimum growth temperature (T_opt_) and maximum mycelial growth rate (*p* < 0.0001 for both variables; Table 2, Figure 5). All tested isolates were able to grow between 10 and 30 °C. No mycelial growth was observed at 5 or 40 °C, whereas limited growth at 35 °C was recorded only for *C. chrysophilum* isolates CVG 2240 and CVG 2255, *C. alienum* isolate CVG 2563, and *C. nymphaeae* isolate CVG 2470. The estimated T_opt_ ranged from 22.5 °C for *C. godetiae* isolate CVG 2466 to 27.2 °C for *C. nymphaeae* isolate CVG 2470. Both *C. chrysophilum* isolates showed relatively high optimum temperatures (23.9–24.5 °C), while intermediate values were observed for *C. alienum* (23.4 °C), *C. fioriniae* (23.2–23.7 °C), and the second *C. nymphaeae* isolate (CCI 208, 24.9 °C). Maximum mycelial growth rate also differed significantly among isolates. Both *C. chrysophilum* isolates exhibited the highest growth rates, followed by *C. alienum*, *C. nymphaeae* isolate CVG 2470, and *C. fioriniae* isolate CVG 2457, which did not differ significantly from one another (Table 2). Lower MGR values were recorded for *C. nymphaeae* isolate CCI 208, whereas *C. godetiae* isolate CVG 2466 and *C. fioriniae* isolate CVG 2517 exhibited the lowest growth rates and did not differ significantly from one another (Table 2).

### 2.4. Pathogenicity Tests

#### 2.4.1. Pathogenicity Test on Fruit

The eight representative tested isolates induced typical bitter rot symptoms on wounded apple fruits, although lesion development showed marked variability (Figure 6a). Symptoms first appeared at 3 dpi as circular, sunken necrotic lesions that progressively enlarged during incubation and were frequently associated with acervuli producing salmon-colored conidial masses. Significant differences in lesion diameter were detected among isolates at 14 dpi, indicating differences in virulence among the tested isolates. The largest mean lesion diameters were recorded for *C. nymphaeae* isolate CVG 2470 (38.32 ± 6.11 mm), *C. fioriniae* isolate CVG 2517 (38.11 ± 5.71 mm), and *C. nymphaeae* isolate CCI 208 (37.39 ± 7.19 mm), followed by *C. alienum* isolate CVG 2563 (33.92 ± 5.27 mm). In contrast, *C. chrysophilum* isolate CVG 2255 produced substantially smaller lesions (4.72 ± 1.86 mm), while *C. godetiae* isolate CVG 2466 showed the lowest mean lesion diameter (1.04 ± 0.63 mm). On unwounded fruits, symptom development was delayed compared with wounded fruits and varied among isolates (Figure 6b). No symptoms were observed at 7 dpi, whereas the first symptoms appeared at 14 dpi on fruits inoculated with *C. fioriniae* isolates CVG 2457 and CVG 2517 and *C. nymphaeae* isolates CVG 2470 and CCI 208. Symptoms subsequently developed in fruits inoculated with the remaining isolates, confirming their ability to cause infection in the absence of wounding. At the final assessment (28 dpi), significant differences in disease severity were detected among isolates (*p* < 0.05), with DSI values ranging from 20 to 55%. The highest DSI values were recorded for *C. nymphaeae* isolate CVG 2470 (55 ± 2.53%) and *C. fioriniae* isolate CVG 2457 (55 ± 1.90%), followed by *C. nymphaeae* isolate CCI 208 (48 ± 3.16%) and *C. alienum* isolate CVG 2563 (42.22 ± 3.79%), which belonged to the same statistical group. Lower disease severity was observed for *C. chrysophilum* isolates CVG 2240 (30 ± 2.85%) and CVG 2255 (20 ± 1.26%), *C. godetiae* isolate CVG 2466 (20 ± 0.95%), and *C. fioriniae* isolate CVG 2517 (20 ± 0.95%). Control fruits remained symptomless throughout the experiment.

#### 2.4.2. Pathogenicity Test on Leaves

All representative isolates induced typical GLS symptoms on both wounded and unwounded detached apple leaves, although disease severity varied among isolates (Figure 6c). Symptoms first appeared at 5 dpi as small necrotic spots that progressively enlarged, became surrounded by chlorotic areas, and occasionally coalesced during incubation. Acervuli were observed on lesions induced by isolates CVG 2240 and CVG 2255 at 10 dpi. At the final assessment (10 dpi), disease severity significantly differed among isolates on wounded leaves. The highest DSI values were recorded for *C. chrysophilum* isolates CVG 2255 (74 ± 7.33%) and CVG 2240 (66 ± 7.33%), followed by *C. nymphaeae* isolate CCI 208 (48 ± 10.41%), *C. godetiae* isolate CVG 2466 (42 ± 8.67%), *C. fioriniae* isolate CVG 2457 (36 ± 13.60%), *C. alienum* isolate CVG 2563 (36 ± 7.78%), and *C. fioriniae* isolate CVG 2517 (32 ± 8.00%). *Colletotrichum nymphaeae* isolate CVG 2470 showed the lowest disease severity (22 ± 11.72%). On unwounded leaves, all isolates were also able to induce GLS symptoms in the absence of wounding. Significant differences in disease severity were detected among isolates at 10 dpi (*p* < 0.05), with the highest DSI values recorded for *C. chrysophilum* isolates CVG 2240 and CVG 2255 (52 ± 11.62% and 52 ± 7.43%, respectively), followed by *C. godetiae* isolate CVG 2466 (40 ± 10.33%) and *C. alienum* isolate CVG 2563 (36 ± 12.22%). The remaining isolates showed lower disease severity, with DSI values ranging from 20 to 32%. Control leaves remained symptomless for both inoculation conditions.

Fungi reisolated from symptomatic fruit and leaf tissues were morphologically consistent with the inoculated isolates, and sequencing of the ApMat (*C. gloeosporioides* SC) or *his3* (*C. acutatum* SC) loci confirmed their identity, thereby fulfilling Koch’s postulates.

### 2.5. In Vitro Fungicide Sensitivity Assays

Sensitivity to the three evaluated fungicides varied among *Colletotrichum* isolates (Table S5, Figure 7). For fludioxonil (Figure 7a), EC_50_ values ranged from 0.002 to 0.073 µg mL^−1^, with the lowest and highest values recorded for isolates CVG 2559 and CVG 3118, respectively. Most isolates showed EC_50_ values below 0.02 µg mL^−1^, whereas only five isolates displayed values >0.04 µg mL^−1^. Isolates within the *C. gloeosporioides* SC showed a broader EC_50_ distribution and included all isolates with the highest values, whereas isolates of the *C. acutatum* SC showed a narrower distribution, with all EC_50_ values ≤ 0.02 µg mL^−1^. For mefentrifluconazole (Figure 7b), EC_50_ values showed a relatively narrow distribution among isolates, with most values ranging from 0.03 to 0.10 µg mL^−1^. Four isolates showed EC_50_ values between 0.12 and 0.13 µg mL^−1^, while isolates CVG 3155 (EC_50_ = 0.158 µg mL^−1^) and CVG 3134 (EC_50_ = 0.207 µg mL^−1^) displayed the highest values within the tested population. Both isolates were identified as members of the *C. gloeosporioides* SC. Overall, isolates of the *C. acutatum* SC showed EC_50_ values below 0.10 µg mL^−1^, whereas those of the *C. gloeosporioides* SC encompassed a broader range and included the isolates with the highest values. For pyraclostrobin (Figure 7c), EC_50_ values showed a broad distribution, ranging from 0.0001 µg mL^−1^ for isolate CVG 3163 to 1.722 µg mL^−1^ for isolate CVG 2253, spanning approximately four orders of magnitude. Most isolates showed low EC_50_ values, whereas four isolates (CVG 2253, CVG 2490, CVG 2249, and CCI 202) displayed values above 1.3 µg mL^−1^. These four isolates were identified as members of the *C. gloeosporioides* SC. Isolates within the *C. acutatum* SC showed consistently low and narrowly distributed EC_50_ values, whereas those within the *C. gloeosporioides* SC spanned the entire EC_50_ range and included all the isolates with the highest values.

## 3. Discussion

The present study provides an integrated characterization of apple-associated *Colletotrichum* isolates collected from commercial orchards in Piedmont, Northwestern Italy, combining taxonomic identification with biological characterization, pathogenicity assessment, and fungicide-sensitivity profiling. Five species belonging to the *C. gloeosporioides* and *C. acutatum* SCs were identified by multilocus phylogenetic analyses of 49 representative isolates selected from the 155 isolates collected from symptomatic leaves and fruits between 2023 and 2025: *C. chrysophilum*, *C. alienum*, *C. fioriniae*, *C. godetiae* and *C. nymphaeae*. *Colletotrichum chrysophilum* was the most represented species across the isolates included in the multilocus phylogeny. This finding is consistent with previous surveys conducted in Northern Italy, where *C. chrysophilum* was reported as the most frequently isolated species associated with GLS and ABR [11], and further supports earlier reports of this species on apple in Italy [12] as well as its occurrence in other apple-growing regions worldwide, including Brazil, Spain and the USA [23,30,31,32,33]. Its consistent recovery across different years, orchards, cultivars, and plant organs suggests that *C. chrysophilum* currently plays a major role in the development of GLS and ABR in the region. However, the biological and ecological factors underlying the apparent success of this species remain largely unknown and deserve further investigation. The recent report of *C. alienum* and *C. nymphaeae* further expands the diversity of *Colletotrichum* species currently known to be associated with apple in Italy [19].

Reliable identification of *Colletotrichum* species required a multilocus phylogenetic approach based on seven loci for the *C. gloeosporioides* SC and five loci for the *C. acutatum* SC. This approach further highlights the limitations of single-locus identification for species delimitation within this genus. Preliminary BLASTn searches based on individual loci frequently suggest affinity with *C. fructicola*, a species that attracted considerable phytosanitary attention in Europe and was the subject of a pest categorization by the European Food Safety Authority (EFSA) due to its potential impact on apple and other hosts [34]. However, multilocus phylogenetic analyses consistently reassigned these isolates to *C. chrysophilum*, demonstrating that species identification based solely on individual loci may lead to inaccurate taxonomic assignments within the *C. gloeosporioides* SC. Our phylogenetic analyses corroborate previous studies highlighting the importance of informative loci for *Colletotrichum* systematics [35,36], with ApMat and *gs* displaying the highest numbers of unique site patterns and providing the greatest phylogenetic resolution within the *C. gloeosporioides* SC, whereas *gapdh*, *tub2*, and *his3* contributed most to species resolution within the *C. acutatum* SC.

Field observations during the three-year survey indicated differences in the occurrence of GLS and ABR among years and orchards. Although disease incidence and severity were not quantitatively assessed, these observations coincided with markedly different climatic conditions across the study period. The first significant outbreaks of GLS observed during 2023 were mainly restricted to a limited number of orchards and coincided with localized periods of increased rainfall during the growing season, particularly in May and August, which may have favored infection and inoculum dispersal [37]. In contrast, both GLS and ABR became widespread throughout the surveyed area during 2024. The widespread occurrence of symptoms observed during the 2024 surveys coincided with exceptionally wet climatic conditions across Piedmont, where annual precipitation reached 1495.7 mm (+45% relative to the 1991–2020 average), making 2024 the second wettest year in the regional historical series [38]. In contrast, symptoms occurrence appeared lower during 2025, despite annual temperatures remaining approximately 1 °C above the climatic average, possibly because of less favorable rainfall distribution and prolonged dry periods during the growing season [39]. Temperature and moisture are recognized as key drivers of GLS and ABR epidemics [40,41,42]. In particular, rainfall events not only favor *Colletotrichum* infection through prolonged wetness periods but also promote splash dispersal of conidia from infected tissues to susceptible organs, thereby contributing to epidemic development [14,43,44,45]. The contrasting disease scenarios observed during the three years of this survey therefore suggest that the availability of moisture, together with suitable temperatures, may have been a major factor influencing the emergence and spread of GLS and ABR in Northwestern Italy. In particular, the transition from localized outbreaks in 2023 to widespread disease in 2024 coincided with climatic conditions considered favorable for *Colletotrichum* infection and inoculum dispersal. These observations highlight the importance of temperature for the development of *Colletotrichum* spp. associated with apple, as differences in isolates may ultimately influence their epidemiology and field distribution [41,42,46]. Although all isolates showed optimum growth within the mesophilic temperature range (22.5–27.2 °C), statistically significant differences in optimum temperature were detected, with *C. nymphaeae* isolate CVG 2470 exhibiting the highest optimum temperature and *C. godetiae* isolate CVG 2466 the lowest. More pronounced differences emerged in maximum growth rate, with the two tested *C. chrysophilum* isolates exhibiting higher maximum growth rates than the other tested isolates. Although the limited number of isolates examined precludes broad ecological generalizations, the consistently higher growth rates observed for *C. chrysophilum* isolates are noteworthy, and raise the possibility that this physiological trait may contribute to its ecological performance. Further studies, including a larger number of isolates, are required to determine whether this physiological trait translates into an ecological advantage under field conditions and whether temperature-dependent growth characteristics contribute to species distribution and seasonal disease development.

Pathogenicity assays confirmed that all representative isolates were able to induce typical disease symptoms on apple tissues and fulfil Koch’s postulates, although marked differences in virulence were observed among isolates. On fruits, the tested isolates of *C. fioriniae*, *C. nymphaeae*, and *C. alienum* generally showed the highest virulence, although their response varied according to the inoculation method. A consistent pattern emerged between wounded and unwounded fruits, with *C. nymphaeae* isolates CVG 2470 and CCI 208 and *C. alienum* isolate CVG 2563 showing high disease severity under both inoculation conditions. In contrast, the two *C. fioriniae* isolates showed different responses depending on the inoculation method, with CVG 2517 showing higher virulence on wounded fruits and CVG2457 on unwounded fruits. The ability of these isolates to induce disease on unwounded fruits confirmed that artificial wounding was not required for infection. However, symptom development was slower on unwounded fruits, consistent with previous reports that wounding facilitates infection by apple-associated *Colletotrichum* spp. [11]. A different pattern emerged on leaves, where the two tested *C. chrysophilum* isolates (CVG 2240 and CVG2255) showed the highest disease severity index under both wounded and unwounded conditions. Tissue-associated differences in the infection behavior of apple-associated *Colletotrichum* isolates have also been recently reported, including preferential appressoria formation by *C. chrysophilum* on apple leaves [47]. The contrasting responses observed on fruits and leaves further suggest that virulence may vary not only at isolate level, but also according to the infected tissue, although a larger number of isolates per species is required to investigate this hypothesis.

The fungicide sensitivity analyses provide valuable information on the response of apple-associated *Colletotrichum* isolates from Northwestern Italy to fungicides currently used in apple cultivation [20,27]. The isolates examined in this study originated from commercial apple orchards. In conventional apple production, fungicides with different modes of action are applied throughout the growing season against different pathogens. Although the active ingredients evaluated in this study are not exclusively used for GLS and ABR management, their application against other apple pathogens may expose *Colletotrichum* to these fungicides. Although detailed fungicide-treatment histories were not available for individual orchards, characterizing the range and distribution of sensitivity within the current isolate collection is therefore relevant for future monitoring, particularly because variation in fungicide sensitivity and resistance to single-site fungicides have already been documented in apple-associated *Colletotrichum* isolates from different production areas worldwide [21,23,25,26,48]. The three tested active ingredients showed distinct sensitivity profiles within the isolate collection. Fludioxonil was characterized by generally low and narrowly distributed EC_50_ values, consistent with previous results obtained for apple-associated *Colletotrichum* [25,32]. Martin et al. [25] reported a low overall fludioxonil EC_50_ (0.029 µg mL^−1^) among isolates causing apple bitter rot in the Mid-Atlantic United States, although sensitivity differed between species complexes. In this study, isolates assigned to the *C. acutatum* SC similarly showed a narrow distribution of low EC_50_ values, whereas greater variability was observed within the *C. gloeosporioides* SC. Differences in fludioxonil response between these species complexes have also been described by Schnabel et al. [49], who further characterized their sensitivity by evaluating the response to increasing fludioxonil concentrations. Mefentrifluconazole also showed low EC_50_ values, with a continuous distribution and no clearly separated group of isolates with elevated EC_50_ values. Information specifically addressing mefentrifluconazole sensitivity in apple associated *Colletotrichum* remains limited. Variation in sensitivity to this active ingredient among ABR isolates has been previously reported, and variation in mefentrifluconazole sensitivity has also been documented in *Colletotrichum* spp. from other pathosystems [25,50]. Pyraclostrobin showed a wide EC_50_ range. Most isolates were strongly inhibited at low concentrations; however, a small number of isolates showed comparatively lower sensitivity. These isolates warrant further investigation to clarify the biological significance of their EC_50_ values. This is particularly relevant for QoI fungicides, as variation in pyraclostrobin sensitivity and resistance have been previously reported in apple-associated *Colletotrichum*, and the G143A substitution in the mitochondrial cytochrome *b* (*cytb*) gene has been identified as an important resistance marker [21,23,47]. In general, the consistently low EC_50_ values observed in this study indicate a high in vitro sensitivity of the tested isolates to fludioxonil, mefentrifluconazole, and pyraclostrobin, while differences in EC_50_ distribution may provide an initial reference for future monitoring of sensitivity shifts in the field.

This study provides an integrated characterization of the *Colletotrichum* species associated with GLS and ABR in Northwestern Italy. The diversity of species found, together with the biological variability observed among isolates in growth characteristics and virulence, emphasizes the complexity of these emerging diseases. The sensitivity profiles obtained for fludioxonil, mefentrifluconazole, and pyraclostrobin provide relevant information for monitoring fungicide response, with the heterogeneous pyraclostrobin distribution warranting particular attention and further molecular investigation. Continuous field monitoring is essential to clarify disease epidemiology, identify the main sources of inoculum, investigate cultivar susceptibility, and determine how disease occurrence and species composition vary over time and in response to environmental conditions and disease-management practices. A better understanding of these aspects, building on the biological and fungicide-sensitivity data generated here, is necessary to develop more precise and effective management strategies for GLS and ABR in apple production.

## 4. Materials and Methods

### 4.1. Sample Collection and Fungal Isolation

Between 2023 and 2025, symptomatic apple leaves and fruits showing typical necrotic lesions associated with GLS and ABR were collected from 28 commercial orchards located in the Piedmont region, Northwestern Italy. Most surveyed orchards were located in the province of Cuneo, the major apple-growing area of Piedmont, while additional orchards from other production areas of the region were also included (Table S1). Orchards were selected based on the occurrence or reporting of symptoms consistent with GLS and/or ABR and their accessibility for sampling. Within each orchard, symptomatic leaves and fruits were collected from different sampling points across the orchard. Samples were obtained from different apple cultivars grown under either conventional or organic management systems; specifically, three orchards were managed organically, whereas the remaining orchards followed conventional practices. Small tissue fragments (5–10 mm) were excised from the area between healthy and necrotic tissues, surface-disinfected in 1% sodium hypochlorite for 1 min, rinsed in sterile distilled water (SDW) for 1 min, and air-dried on sterile absorbent paper. Subsequently, smaller fragments (2–3 mm) were plated onto potato dextrose agar (PDA; VWR Chemicals, Leuven, Belgium) amended with streptomycin sulphate (25 mg L^−1^; Sigma-Aldrich, St. Louis, MO, USA) (PDA-S). Plates were incubated at 25 ± 1 °C under a 12 h photoperiod for 48–72 h. Hyphal tips taken from the margins of actively growing colonies were transferred onto fresh PDA-S plates to obtain pure cultures. A collection of 155 *Colletotrichum*-like isolates was obtained. The isolates CVG 2563 and CVG 2470 had previously been reported as *C. alienum* and *C. nymphaeae*, respectively, in association with apple in Italy [19]. Both isolates were retained in the present study for comparative phylogenetic and biological characterization. All isolates are currently preserved at −80 °C in the Plant Pathology Culture Collection of the University of Torino (Torino, Italy). In addition, eight representative isolates were deposited in the Turin University Culture Collection (TUCC), Torino, Italy.

### 4.2. DNA Extraction, Polymerase Chain Reaction (PCR) Amplification, and Sanger Sequencing

The total genomic DNA was extracted from all 155 isolates using the E.Z.N.A.^®^ Fungal DNA Mini Kit (Omega Bio-Tek, Darmstadt, Germany) according to the manufacturer’s instructions. To assign the isolates to the major *Colletotrichum* SCs, PCR amplification and sequencing of the glyceraldehyde-3-phosphate dehydrogenase (*gapdh*) locus was performed for all the obtained isolates. Based on the *gapdh* phylogeny, 37 representative isolates from the *C. gloeosporioides* SC and 12 from the *C. acutatum* SC were selected for multilocus phylogenetic analyses for species-level identification. Isolates were chosen according to colony morphology, host tissue, cultivar, sampling year, geographical location origin, and their placement within the *gapdh* phylogenetic clusters, with the aim of representing the phenotypic, geographical and genetic diversity observed among the collected isolates. Species-level identification was carried out by sequencing additional loci, including chitin synthase 1 (*chs-1*), actin (*act*), β-tubulin (*tub2*), calmodulin (*cal*), glutamine synthetase (*gs*), histone H3 (*his3*), and the Apn2-Mat1-2 intergenic spacer and partial mating type gene (ApMat). Partial *gapdh* sequences were amplified using primer pair GDF1/GDR1 [51], whereas amplification of *chs-1* and *act* loci was performed using primers CHS-79F/CHS-345R and ACT-512F/ACT-783R, respectively [52]. Partial *tub2*, *cal*, and *his3* genes were amplified using primer pairs T1/Bt2b [53,54], CL1C/CL2C [55], and CYLH3F/CYLH3R [56], respectively. Amplification of *gs* and ApMat loci was carried out using primer pairs GSF1/GSR1 and AMF1/AMR1, respectively [35]. PCR mixtures and thermal cycling conditions for all loci followed the protocols described by Guarnaccia et al. [57] and Liu et al. [35]. Amplification products were evaluated by electrophoresis on 1% agarose gels (VWR Life Science AMRESCO^®^ Biochemicals, Solon, OH, USA) stained with GelRed™ (Biotium, Fremont, CA, USA). PCR products were sequenced in both directions by Macrogen Europe (Milan, Italy). Raw chromatograms were assembled, trimmed, and analysed using Geneious Prime^®^ version 11.0.20.1+1 (Biomatters Ltd., Auckland, New Zealand).

### 4.3. Phylogenetic Analyses

Sequences generated in this study were initially compared with those available in the National Center for Biotechnology Information (NCBI) GenBank database using the BLASTn algorithm (https://blast.ncbi.nlm.nih.gov/, accessed on 14 January 2026) to obtain preliminary species identification. Based on the multilocus dataset, phylogenetic analyses were conducted separately for isolates belonging to the *C. gloeosporioides* and *C. acutatum* SCs. For the *C. gloeosporioides* SC, a combined dataset of 120 strains, including 83 reference strains retrieved from GenBank and 37 isolates from the present collection, including the previously reported isolate CVG 2563 [19], was assembled using sequences from seven loci: *gapdh*, *chs-1*, *act*, *tub2*, *cal*, *gs*, and ApMat. For the *C. acutatum* SC, phylogenetic analyses included a total of 67 strains, consisting of 55 reference strains retrieved from GenBank, and 12 isolates from the present collection, including the previously reported isolate CVG 2470 [19], based on five loci: *gapdh*, *chs-1*, *his3*, *act*, and *tub2*. Ex-type and epitype strains were included in both datasets whenever available. *Colletotrichum pyrifoliae* (CGMCC 3.18902) and *C. orchidophilum* (CBS 632.80) were selected as outgroups for the *C. gloeosporioides* and *C. acutatum* SCs, respectively, according to [36]. Sequences for each locus were aligned independently using MAFFT v.7 (https://mafft.cbrc.jp/alignment/server/, accessed on 18 March 2026) and concatenated in MEGA v.7 [58]. Phylogenetic relationships were inferred using both Maximum Parsimony (MP) and Bayesian Inference (BI) approaches. Maximum Parsimony analyses were performed in PAUP v.4.0a169 [59] using heuristic search algorithms with 100 random taxon addition replicates and tree bisection-reconnection (TBR) branch swapping. All characters were treated with equal weight, and phylogenetic support was evaluated through bootstrap analyses based on 1000 replicates. Standard phylogenetic parameters, including tree length (TL), consistency index (CI), retention index (RI), and rescaled consistency index (RC), were calculated for the resulting trees. Bayesian Inference analyses were conducted using MrBayes v.3.2.7 [60] and the best-fit nucleotide substitution model for each locus was selected with MrModeltest v.2.3 [61]. Markov Chain Monte Carlo (MCMC) simulations were performed with four simultaneous chains starting from random trees, with trees sampled every 1000 generations. Runs were continued until an average standard deviation of split frequencies below 0.01 was achieved. Final phylogenetic trees were visualized using FigTree v.1.4.

### 4.4. Effect of Temperature on Mycelial Growth

Eight representative isolates (CVG 2240 and CVG 2255—*C. chrysophilum*, CVG 2457 and CVG 2517—*C. fioriniae*, CVG 2466—*C. godetiae*, CVG 2470 and CCI 208—*C. nymphaeae*, and CVG 2563—*C. alienum*) were selected for temperature-dependent mycelial growth analyses. Isolates were grown on PDA-S at 25 ± 1 °C in the dark for 7 days. Mycelial plugs (5 mm diameter) taken from the margin of actively growing colonies were transferred onto the center of fresh PDA-S plates, incubated at 5, 10, 15, 20, 25, 30, 35 and 40 °C in the dark and monitored daily. Four replicates were used for each isolate-temperature combination in a completely randomized experimental design. Colony diameters were measured along two perpendicular axes using a ruler 5 days after inoculation. Mean values were converted into radial growth rates (mm day^−1^). The relationship between temperature and mycelial growth was analyzed by fitting the generalized Analytis Beta model [62] as described by López-Moral et al. [63]. Regression curves obtained for each isolate were used to estimate minimum, optimum, and maximum growth temperatures, as well as the maximum growth rate (MGR). Data were tested for normality and homogeneity of variances prior to statistical analyses. Analysis of variance (ANOVA) was performed considering optimum growth temperature and MGR as dependent variables and isolates as independent variables. Mean comparisons were conducted using Tukey’s honestly significant difference (HSD) test at *p* ≤ 0.05. Statistical analyses were performed using Statistix 10 software (Analytical Software, Tallahassee, FL, USA) [63].

### 4.5. Pathogenicity Tests

Two independent pathogenicity experiments were conducted on detached apple fruits and leaves of cv. Gala using representative isolates previously selected for temperature-dependent growth analyses. For each isolate, a conidial suspension adjusted to 1 × 10^5^ conidia mL^−1^ was prepared. SDW as used as negative control. To fulfil Koch’s postulates, fungi were reisolated from symptomatic tissues at the end of each experiment, and their identity was confirmed by sequencing the ApMat locus for isolates belonging to the *C. gloeosporioides* SC and the *his3* locus for isolates belonging to the *C. acutatum* SC.

#### 4.5.1. Pathogenicity Test on Fruits

Pathogenicity on detached apple fruits was evaluated using both wounded and unwounded inoculation methods. A total of 216 fruits were used, corresponding to 12 fruits per isolate and control treatment for each inoculation method. Regarding wounded inoculations, fruits were punctured at three equidistant sites using a sterile needle, and 20 μL drops of conidial suspension (1 × 10^5^ conidia mL^−1^) was deposited onto each wound. In unwounded inoculations, 1 mL of conidial suspension was uniformly sprayed onto the fruit surface. Control fruits received SDW following the same inoculation procedures. After inoculation, fruits were placed in humid chambers and incubated at 25 ± 1 °C under a 12-h photoperiod. Fruits were inspected daily for symptom development. On wounded fruits, lesion size was assessed at 14 days post inoculation (dpi) by measuring lesion diameters along two perpendicular axes and calculating the mean lesion diameter. On unwounded fruits, symptom development was evaluated at 14, 21, and 28 dpi, while disease severity recorded at the final assessment (28 dpi) was used for statistical analyses. Disease severity was assessed using a semi-quantitative ordinal scale based on the estimated percentage of symptomatic fruit surface: 0 = no symptoms; 1 = 1–10%; 2 = 11–25%; 3 = 26–45%; 4 = 46–69%; and 5 = >70% symptomatic fruit surface. Disease severity scores were used to calculate the Disease Severity Index (DSI) according to the following formula: DSI (%) = [Σ(n × v)/(N × V)] × 100, where n is the number of fruits assigned to each severity class, v is the corresponding severity score, N is the total number of fruits evaluated, and V is the maximum severity score.

#### 4.5.2. Pathogenicity Tests on Leaves

Pathogenicity on detached leaves was evaluated using both wounded and unwounded inoculation methods. For each isolate, 20 leaves were inoculated at three equidistant points with 20 μL drops of conidial suspension (1 × 10^5^ conidia mL^−1^). Half of the leaves were wounded using a sterile needle prior to inoculation, whereas the remaining leaves were left unwounded. Control leaves received SDW following the same inoculation procedures. Inoculated leaves were placed in humid chambers containing moistened sterile paper towels and incubated at 25 ± 1 °C under a 12-h photoperiod. Leaves were inspected daily for symptom development, and disease severity was evaluated at 3, 5 and 10 dpi. Disease severity data recorded at the final assessment (10 dpi) were used for statistical analyses. For both wounded and unwounded leaves, disease severity was estimated using a semi-quantitative ordinal scale based on the percentage of symptomatic leaf area: 0 = no symptoms; 1 = ≤5%; 2 = 6–10%; 3 = 11–25%; 4 = 26–50%; and 5 = 51–100% symptomatic leaf area. A visual reference scale showing representative leaves for each severity class was used throughout the experiment to support consistent score assignment (Figure S1). All disease severity assessments were performed by the same evaluator throughout the experiment using the visual reference scale. The DSI was calculated as described above and it represents a normalized index derived from the ordinal severity scores.

### 4.6. In Vitro Fungicide Sensitivity Assays

The in vitro sensitivity assays were performed on *Colletotrichum* isolates obtained in this study. Three fungicides belonging to different FRAC groups were tested: fludioxonil (Geoxe^®^, Syngenta Crop Protection AG, Basel, Switzerland; FRAC 12), mefentrifluconazole (Revysion^®^, BASF SE, Ludwigshafen, Germany; FRAC 3), and pyraclostrobin (Cabrio^®^ WG, BASF SE, Ludwigshafen, Germany; FRAC 11). Mycelial growth inhibition assays were conducted for fludioxonil and mefentrifluconazole, whilst conidial germination assays were performed for pyraclostrobin, following previously described sensitivity-testing approaches for *Colletotrichum* spp. and according to their mode of action [64,65,66]. Preliminary tests were conducted to establish appropriate fungicide concentrations for the spiral gradient assays. Stock solutions were prepared from the commercial formulations taking into account the concentration of pure active ingredients in each product. Depending on the assay, fungicide solutions of 10 or 50 μg mL^−1^ for fludioxonil, 50 or 100 μg mL^−1^ for mefentrifluconazole, and 1, 5, 10, or 1000 μg mL^−1^ for pyraclostrobin were used to generate the concentration gradients. The Petri dishes used for the spiral gradient dilution method were prepared by pouring 15 mL of PDA into each 90-mm Petri dish to obtain a 3-mm-thick PDA layer. Fungicide suspensions (50 μL) were applied as a continuous concentration gradient onto the agar surface using an Eddy Jet 2W Spiral Plater (IUL S.A.). Plates were maintained at room temperature for 3 to 4 h to allow fungicide diffusion through the medium. For pyraclostrobin, assays were performed on PDA amended with salicylhydroxamic acid (SHAM; 100 μg mL^−1^) to inhibit the alternative oxidase pathway [65]. Since the spiral gradient application started off-center, the central region of each Petri dish remained fungicide-free, allowing potential fungal overgrowth and lateral spread. To avoid this, a 14-mm agar plug was removed from the center of each plate using a sterile cork borer. For mycelial growth assays, four sterile absorbent paper strips colonized by actively growing mycelium of the same isolate were placed radially on each plate, perpendicular to one another and extending across the fungicide gradient. For conidial germination assays, a total of 24 μL of conidial suspension (1 × 10^5^ conidia mL^−1^) was applied at four points along the edge of the agar plate (6 μL per point) and uniformly spread across the gradient surface. One isolate was tested per plate, and three replicate plates were used for each isolate—fungicide combination. Control plates consisted of fungicide-free PDA or PDA amended with SHAM for pyraclostrobin assays. Plates were incubated at 25 ± 1 °C under a 12-h photoperiod for 72 h. Each experiment was conducted twice. For mycelial growth assays, the position along each radial growth front at which mycelial growth was reduced by 50% relative to the corresponding untreated control was identified, and the radial distance from this point to the center of the plate was measured [67]. For conidial germination assays, conidia were examined along the fungicide gradient, and the position at which germination was reduced by 50% relative to the corresponding control was determined and measured [67]. The measured radial distances, together with additional parameters including the molecular weight of the fungicide’s active ingredient, the agar layer thickness and the concentration of the fungicide solution used to generate each gradient, were entered into the ECx R package [68]. These parameters were used to calculate the fungicide concentration corresponding to the measured endpoint and to estimate EC_50_ values for each isolate—fungicide combination. This software was originally developed for trials conducted on 150-mm Petri dishes and was subsequently optimized to account for the 90-mm Petri dishes used in the present study. EC_50_ values were interpreted separately for each active ingredient. EC_50_ values were obtained for 154 isolates for fludioxonil and mefentrifluconazole and for 97 isolates for pyraclostrobin. Pyraclostrobin assays could not be performed for 58 isolates because they did not produce conidia under the experimental conditions.

## Figures and Tables

**Figure 1:**
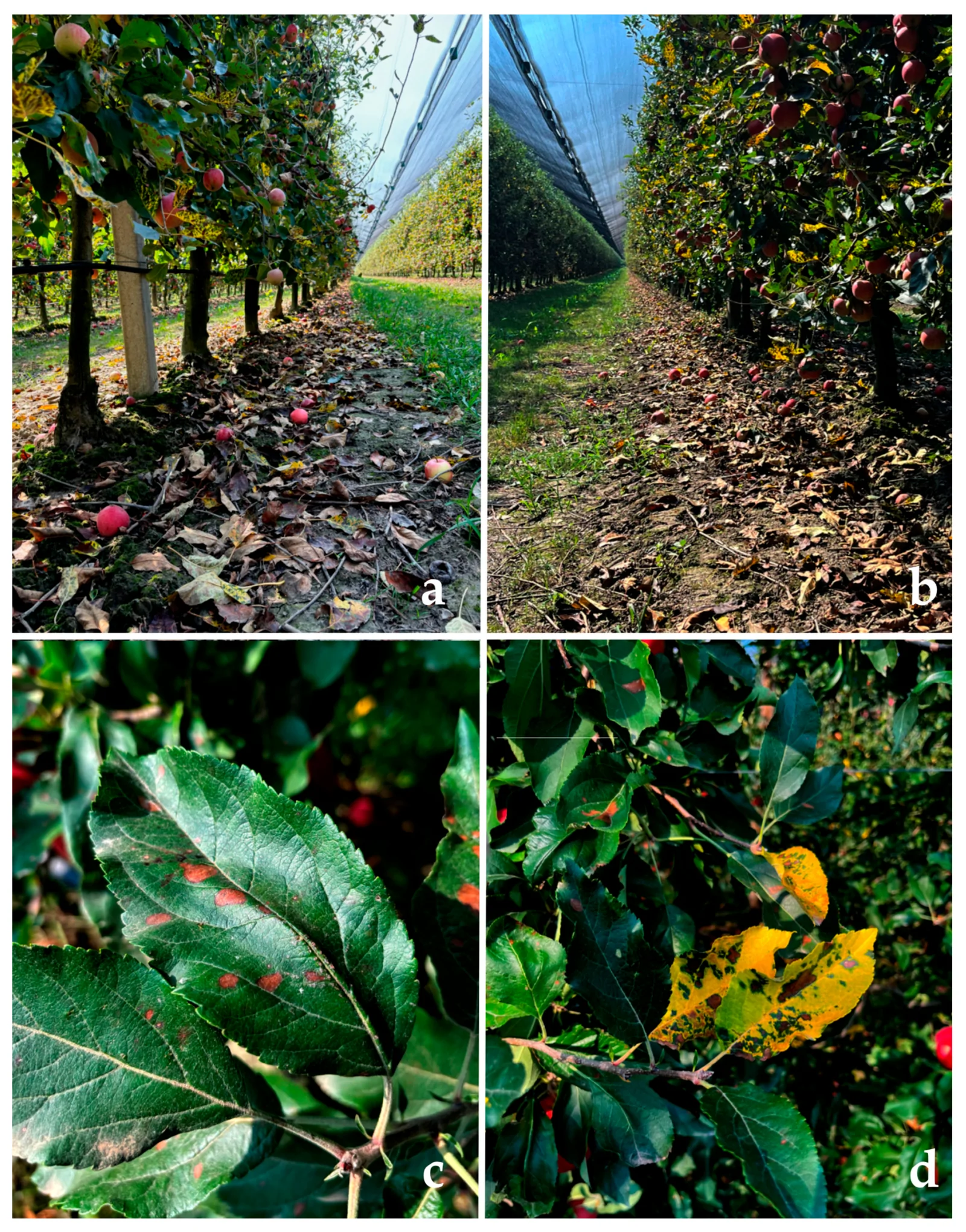
Glomerella leaf spot (GLS) symptoms observed in commercial apple orchards in Northwestern Italy. (**a**,**b**) Severe GLS outbreaks associated with extensive early defoliation and accumulation of fallen leaves beneath affected trees. (**c**,**d**) Typical GLS symptoms on apple leaves, consisting of reddish-brown necrotic spots surrounded by chlorotic tissue, with lesions enlarging and coalescing at advanced stages.

**Figure 2:**
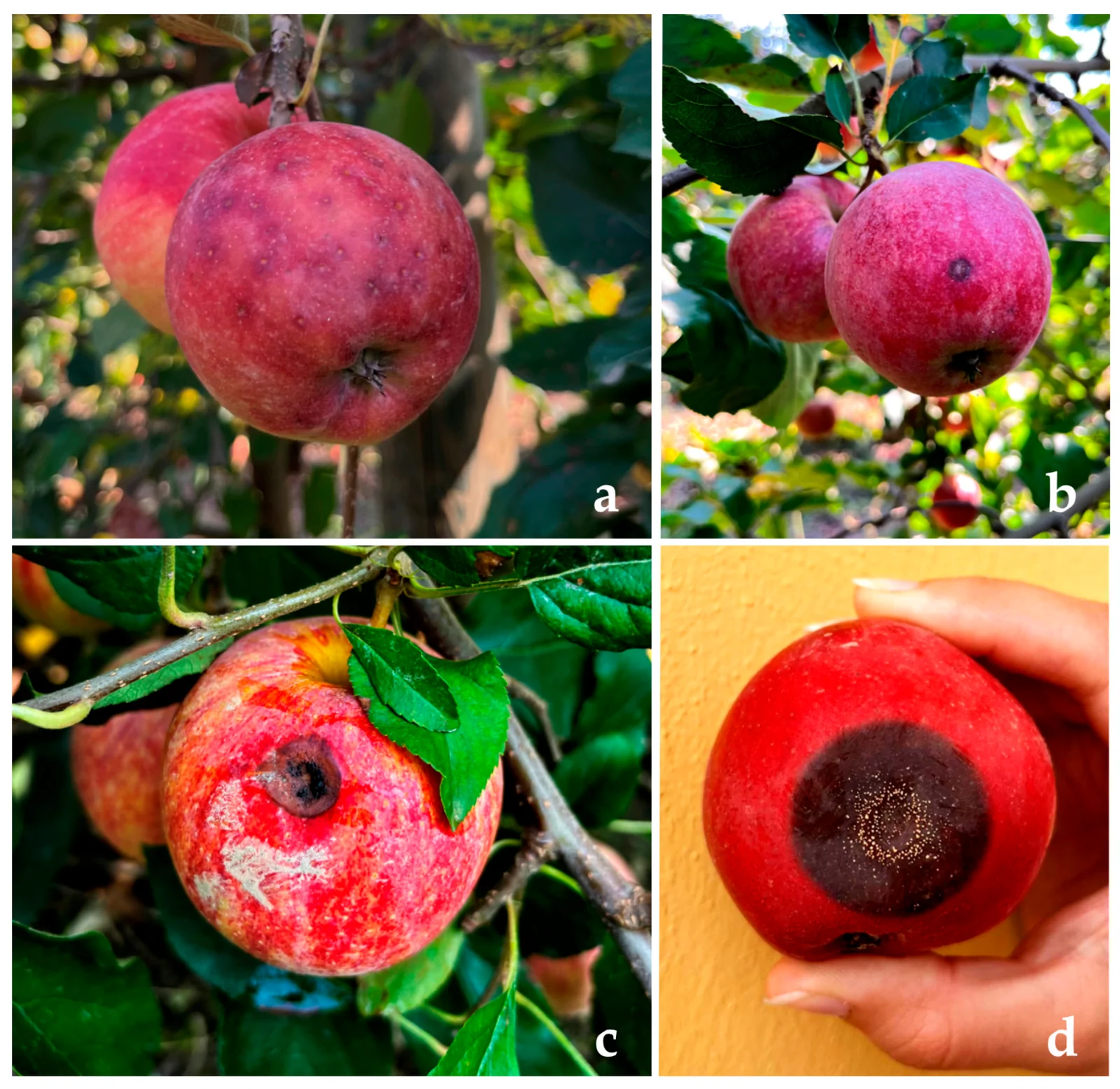
Apple bitter rot (ABR) symptoms observed on apple fruits in commercial orchards in Northwestern Italy. (**a**,**b**) Small, circular, dark brown to black lesions. (**c**,**d**) Advanced ABR symptoms characterized by larger, dark, sunken lesions with acervuli producing salmon-colored conidial masses.

**Figure 3:**
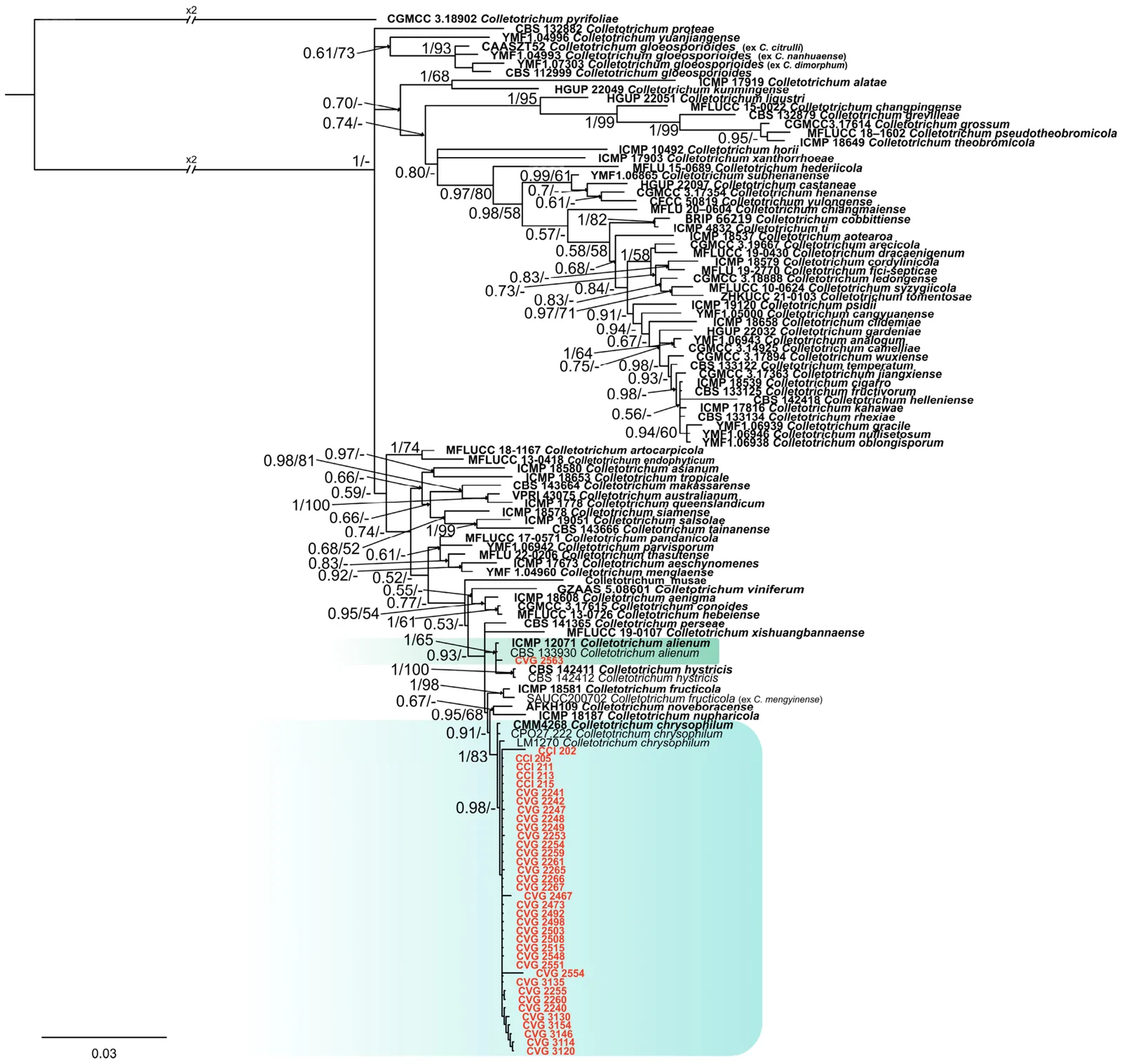
Consensus phylogram of 46,412 trees resulting from a Bayesian analysis of the combined gapdh, chs-1, act, tub2, cal, gs and ApMat sequences of *Colletotrichum gloeosporioides* SC isolates. Bayesian posterior probability values and bootstrap support values are indicated at the nodes. The tree was rooted to *Colletotrichum pyrifoliae* (CGMCC 3.18902). Isolates from the current study are indicated in red.

**Figure 4:**
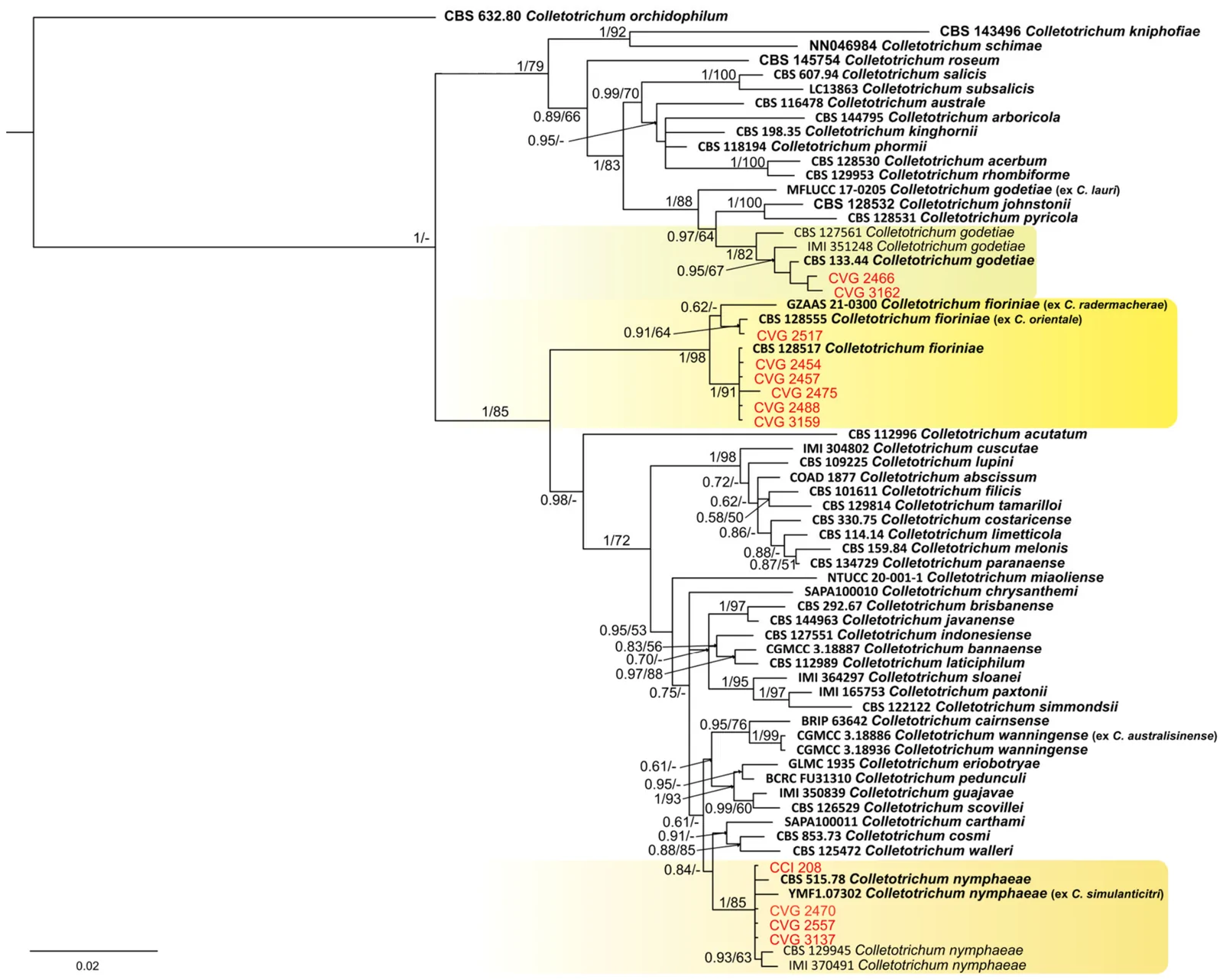
Consensus phylogram of 1.952 trees resulting from a Bayesian analysis of the combined gapdh, chs-1, his3, act and tub2 sequences of *Colletotrichum acutatum* SC isolates. Bayesian posterior probability values and bootstrap support values are indicated at the nodes. The tree was rooted to *Colletotrichum orchidophilum* (CBS 632.80). Isolates from the current study are indicated in red.

**Figure 5:**
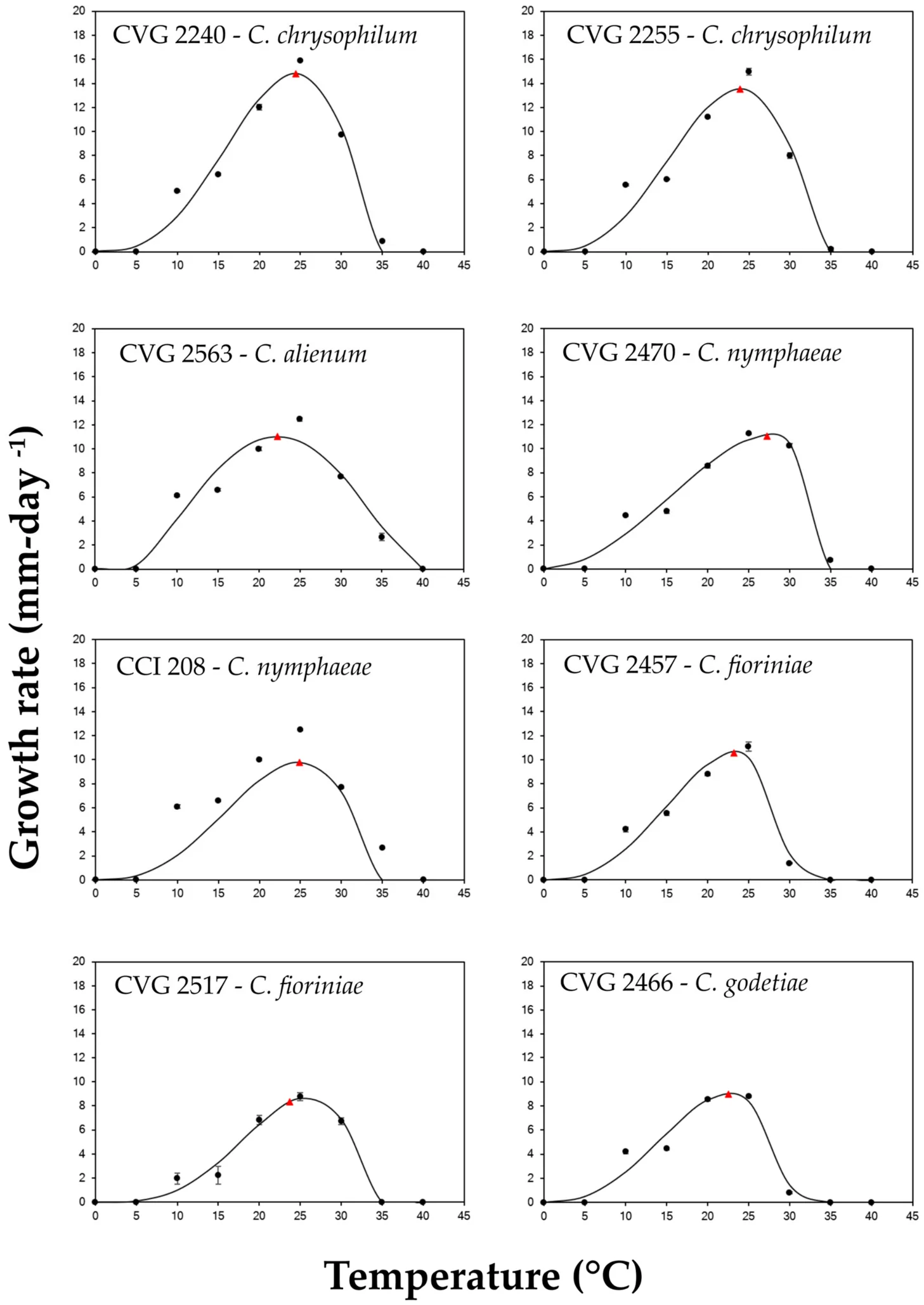
Effect of temperature on mycelial growth rate (mm day ^−1^) of the 8 representative isolates selected in this study grown on PDA at 5, 10, 15, 20, 25, 30, 35 and 40 °C in the dark for 5 days. For each isolate, average growth rates over temperature were adjusted to a nonlinear regression curve using the Analytics Beta model. Data points are the means of four replicated Petri dishes each. Vertical bars are the standard error of the means. The red triangle indicates the estimated optimum growth temperature (T_opt_), corresponding to the maximum growth rate (MGR) predicted by the Analytis Beta model. CVG 2240 and CVG 2255—*C. chrysophilum*, CVG 2563—*C. alienum*, CVG 2470 and CCI 208—*C. nymphaeae*, CVG 2457 and CVG2517—*C. fioriniae* and CVG 2466—*C. godetiae*.

**Figure 6:**
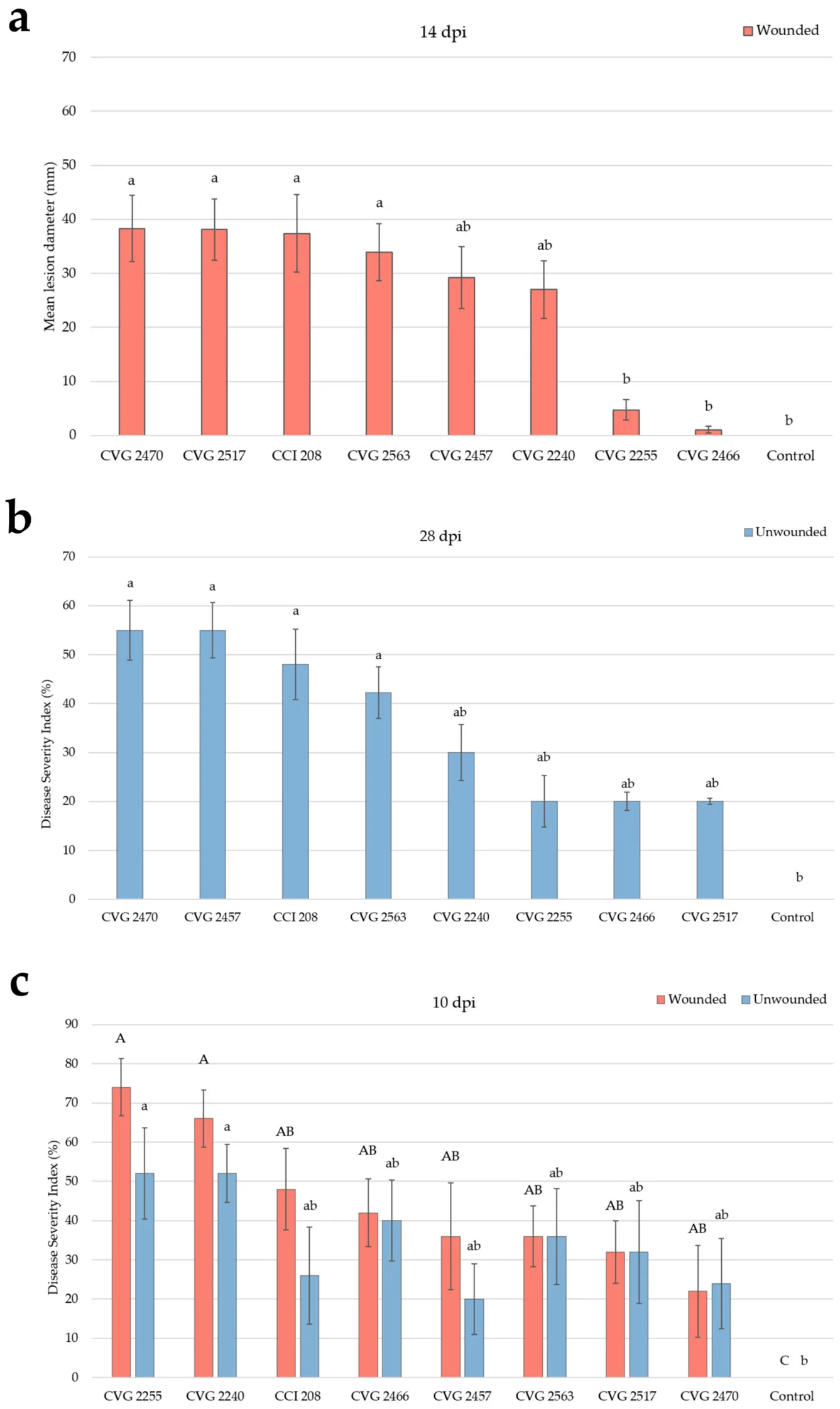
Pathogenicity of representative *Colletotrichum* isolates on detached apple fruits and leaves of ‘Gala’. Isolates included CVG 2240 and CVG 2255 (*C. chrysophilum*), CVG 2457 and CVG 2517 (*C. fioriniae*), CVG 2466 (*C. godetiae*), CVG 2470 and CCI 208 (*C. nymphaeae*), and CVG 2563 (*C. alienum*). (**a**) Mean lesion diameter (mm) on wounded fruits at 14 days post-inoculation (dpi). (**b**) Disease Severity Index (DSI, %) on unwounded fruits at 28 dpi. (**c**) Disease Severity Index (DSI, %) on wounded and unwounded leaves at 10 dpi. DSI (%) represents a normalized index derived from the ordinal severity scores. Bars represent mean values ± standard error (SE). Different letters indicate significant differences between isolates and the control according to Kruskal–Wallis test followed by Dunn’s multiple-comparison test (*p* < 0.05). In panel (**c**), uppercase and lowercase letters indicate statistical comparisons among wounded and unwounded treatments, respectively.

**Figure 7:**
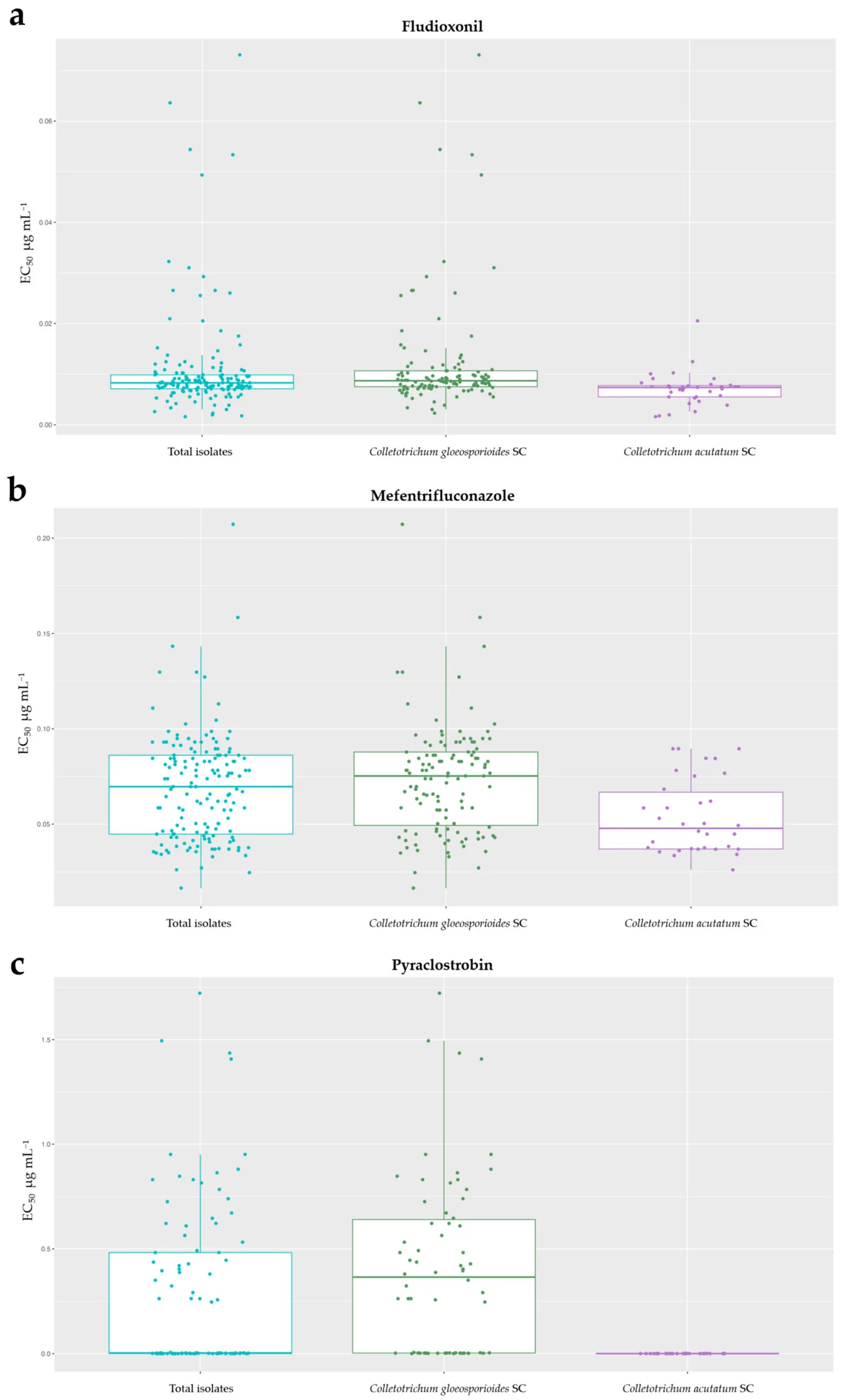
Distribution of EC_50_ values of *Colletotrichum* isolates for (**a**) fludioxonil (*n* = 154), (**b**) mefentrifluconazole (*n* = 154), and (**c**) pyraclostrobin (*n* = 97). Boxplots show EC_50_ distributions for all isolates (light blue) and separately for isolates assigned to the *C. gloeosporioides* (green) and *C. acutatum* (purple) species complexes. Individual points represent single isolates. Boxes indicate the interquartile range and horizontal lines indicate the median.

**Table 1:** *Colletotrichum* isolates selected for multilocus phylogenetic analyses in this study, with species identification and GenBank accession numbers.

CODE ^a^	Species	GenBank Accession Numbers ^b,c^							
		*gapdh*	*chs-1*	*his3*	*act*	*tub2*	*cal*	*gs*	ApMat
CVG 2240–TUCC 1482	*Colletotrichum chrysophilum*	PZ683732	PZ683768	-	PZ683804	PZ683840	PZ683876	PZ683912	PZ683948
CVG 2241	*Colletotrichum chrysophilum*	PZ683707	PZ683743	-	PZ683779	PZ683815	PZ683851	PZ683887	PZ683923
CVG 2242	*Colletotrichum chrysophilum*	PZ683708	PZ683744	-	PZ683780	PZ683816	PZ683852	PZ683888	PZ683924
CVG 2247	*Colletotrichum chrysophilum*	PZ683709	PZ683745	-	PZ683781	PZ683817	PZ683853	PZ683889	PZ683925
CVG 2248	*Colletotrichum chrysophilum*	PZ683710	PZ683746	-	PZ683782	PZ683818	PZ683854	PZ683890	PZ683926
CVG 2249	*Colletotrichum chrysophilum*	PZ683711	PZ683747	-	PZ683783	PZ683819	PZ683855	PZ683891	PZ683927
CVG 2253	*Colletotrichum chrysophilum*	PZ683712	PZ683748	-	PZ683784	PZ683820	PZ683856	PZ683892	PZ683928
CVG 2254	*Colletotrichum chrysophilum*	PZ683713	PZ683749	-	PZ683785	PZ683821	PZ683857	PZ683893	PZ683929
CVG 2255 – TUCC 1483	*Colletotrichum chrysophilum*	PZ683730	PZ683766	-	PZ683802	PZ683838	PZ683874	PZ683910	PZ683946
CVG 2259	*Colletotrichum chrysophilum*	PZ683714	PZ683750	-	PZ683786	PZ683822	PZ683858	PZ683894	PZ683930
CVG 2260	*Colletotrichum chrysophilum*	PZ683731	PZ683767	-	PZ683803	PZ683839	PZ683875	PZ683911	PZ683947
CVG 2261	*Colletotrichum chrysophilum*	PZ683715	PZ683751	-	PZ683787	PZ683823	PZ683859	PZ683895	PZ683931
CVG 2265	*Colletotrichum chrysophilum*	PZ683716	PZ683752	-	PZ683788	PZ683824	PZ683860	PZ683896	PZ683932
CVG 2266	*Colletotrichum chrysophilum*	PZ683717	PZ683753	-	PZ683789	PZ683825	PZ683861	PZ683897	PZ683933
CVG 2267	*Colletotrichum chrysophilum*	PZ683718	PZ683754	-	PZ683790	PZ683826	PZ683862	PZ683898	PZ683934
CVG 2454	*Colletotrichum fioriniae*	PZ669173	PZ669184	PZ669195	PZ669206	PZ669217	-	-	-
CVG 2457 – TUCC 1486	*Colletotrichum fioriniae*	PZ669174	PZ669185	PZ669196	PZ669207	PZ669218	-	-	-
CVG 2466 –TUCC 1485	*Colletotrichum godetiae*	PZ669178	PZ669189	PZ669200	PZ669211	PZ669222	-	-	-
CVG 2467	*Colletotrichum chrysophilum*	PZ683719	PZ683755	-	PZ683791	PZ683827	PZ683863	PZ683899	PZ683935
CVG 2470 – TUCC 1488	*Colletotrichum nymphaeae*	PX966954	PX966955	PX966956	PX966957	PX966958	-	-	-
CVG 2473	*Colletotrichum chrysophilum*	PZ683720	PZ683756	-	PZ683792	PZ683828	PZ683864	PZ683900	PZ683936
CVG 2475	*Colletotrichum fioriniae*	PZ669175	PZ669186	PZ669197	PZ669208	PZ669219	-	-	-
CVG 2488	*Colletotrichum fioriniae*	PZ669176	PZ669187	PZ669198	PZ669209	PZ669220	-	-	-
CVG 2492	*Colletotrichum chrysophilum*	PZ683721	PZ683757	-	PZ683793	PZ683829	PZ683865	PZ683901	PZ683937
CVG 2498	*Colletotrichum chrysophilum*	PZ683722	PZ683758	-	PZ683794	PZ683830	PZ683866	PZ683902	PZ683938
CVG 2503	*Colletotrichum chrysophilum*	PZ683723	PZ683759	-	PZ683795	PZ683831	PZ683867	PZ683903	PZ683939
CVG 2508	*Colletotrichum chrysophilum*	PZ683724	PZ683760	-	PZ683796	PZ683832	PZ683868	PZ683904	PZ683940
CVG 2515	*Colletotrichum chrysophilum*	PZ683725	PZ683761	-	PZ683797	PZ683833	PZ683869	PZ683905	PZ683941
CVG 2517 – TUCC 1487	*Colletotrichum fioriniae*	PZ669172	PZ669183	PZ669194	PZ669205	PZ669216	-	-	-
CCI 202	*Colletotrichum chrysophilum*	PZ683702	PZ683738	-	PZ683774	PZ683810	PZ683846	PZ683882	PZ683918
CCI 205	*Colletotrichum chrysophilum*	PZ683703	PZ683739	-	PZ683775	PZ683811	PZ683847	PZ683883	PZ683919
CCI 208 – TUCC 1489	*Colletotrichum nymphaeae*	PZ669169	PZ669180	PZ669191	PZ669202	PZ669213	-	-	-
CCI 211	*Colletotrichum chrysophilum*	PZ683704	PZ683740	-	PZ683776	PZ683812	PZ683848	PZ683884	PZ683920
CCI 213	*Colletotrichum chrysophilum*	PZ683705	PZ683741	-	PZ683777	PZ683813	PZ683849	PZ683885	PZ683921
CCI 215	*Colletotrichum chrysophilum*	PZ683706	PZ683742	-	PZ683778	PZ683814	PZ683850	PZ683886	PZ683922
CVG 2548	*Colletotrichum chrysophilum*	PZ683726	PZ683762	-	PZ683798	PZ683834	PZ683870	PZ683906	PZ683942
CVG 2551	*Colletotrichum chrysophilum*	PZ683727	PZ683763	-	PZ683799	PZ683835	PZ683871	PZ683907	PZ683943
CVG 2554	*Colletotrichum chrysophilum*	PZ683728	PZ683764	-	PZ683800	PZ683836	PZ683872	PZ683908	PZ683944
CVG 2557	*Colletotrichum nymphaeae*	PZ669170	PZ669181	PZ669192	PZ669203	PZ669214	-	-	-
CVG 2563 – TUCC 1484	*Colletotrichum alienum*	PX966959	PX966960	-	PX966961	PX966962	PX966963	PX966964	PX966965
CVG 3114	*Colletotrichum chrysophilum*	PZ683736	PZ683772	-	PZ683808	PZ683844	PZ683880	PZ683916	PZ683952
CVG 3120	*Colletotrichum chrysophilum*	PZ683737	PZ683773	-	PZ683809	PZ683845	PZ683881	PZ683917	PZ683953
CVG 3130	*Colletotrichum chrysophilum*	PZ683733	PZ683769	-	PZ683805	PZ683841	PZ683877	PZ683913	PZ683949
CVG 3135	*Colletotrichum chrysophilum*	PZ683729	PZ683765	-	PZ683801	PZ683837	PZ683873	PZ683909	PZ683945
CVG 3137	*Colletotrichum nymphaeae*	PZ669171	PZ669182	PZ669193	PZ669204	PZ669215	-	-	-
CVG 3146	*Colletotrichum chrysophilum*	PZ683735	PZ683771	-	PZ683807	PZ683843	PZ683879	PZ683915	PZ683951
CVG 3154	*Colletotrichum chrysophilum*	PZ683734	PZ683770	-	PZ683806	PZ683842	PZ683878	PZ683914	PZ683950
CVG 3159	*Colletotrichum fioriniae*	PZ669177	PZ669188	PZ669199	PZ669210	PZ669221	-	-	-
CVG 3162	*Colletotrichum godetiae*	PZ669179	PZ669190	PZ669201	PZ669212	PZ669223	-	-	-

^a^ CVG and CCI: Plant Pathology Culture Collection of the University of Torino (Torino, Italy); TUCC: Turin University Culture Collection (TUCC), Torino, Italy. ^b^ *gapdh*: glyceraldehyde-3-phosphate dehydrogenase region; *chs-1*: chitin synthase region; *his3*: histone H3 region; *act*: actin region; *tub2*: beta-tubulin region; *cal*: calmodulin gene; *gs*: glutamine synthetase region; ApMat: Apn2-Mat1-2 intergenic spacer and partial mating type gene; ^c^ “-” indicates not analyzed or missing data.

**Table 2:** Effect of temperature on mycelial growth of the representative fungal isolates selected in this study grown on PDA-S at 5, 10, 15, 20, 25, 30, 35 and 40 °C in darkness after 5 days ^v^.

Fungal Species	Isolate	Analytis Beta Model ^w^			Temperature (°C) ^x,z^			MGR (mm day^−1^) ^y,z^
		*R* ^2^	a	b	Optimum	Minimum	Maximum	
*Colletotrichum chrysophilum*	CVG 2240	0.955	3.09	1.32	24.5 bc	0	35.0	14.8 a
*Colletotrichum chrysophilum*	CVG 2255	0.929	3.03	1.40	23.9 cd	0	35.0	13.5 b
*Colletotrichum alienum*	CVG 2563	0.912	2.32	1.48	23.4 de	0	38.0	11.3 c
*Colletotrichum nymphaeae*	CVG 2470	0.960	1.98	0.57	27.2 a	0	35.0	11.1 c
*Colletotrichum nymphaeae*	CCI 208	0.953	2.87	1.17	24.9 b	0	35.0	9.8 d
*Colletotrichum fioriniae*	CVG 2457	0.940	2.70	0.85	23.2 e	0	30.5	10.5 c
*Colletotrichum fioriniae*	CVG 2517	0.971	3.62	1.28	23.7 de	0	35.0	8.7 e
*Colletotrichum godetiae*	CVG 2466	0.925	2.65	0.94	22.5 f	0	30.5	9.0 de

^v^ Data represents the average of four replicated Petri dishes per isolate and temperature combination. ^w^ Analytis Beta model, where *R*^2^ = coefficient of determination, and a, b = coefficients of regression. ^x^ For each isolate, temperature average growth rates were adjusted to a regression curve to estimate the optimum growth temperature. ^y^ MGR: Maximum growth rate (mm per day) obtained by the Analytis Beta model at the optimum growth temperature. ^z^ Means in a column followed by the same letter do not differ significantly according to Tukey’s HSD test at *p* < 0.0001.

## Data Availability

Data are contained within the article and Supplementary Materials.

## Supplementary Materials

The following supporting information can be downloaded at: https://www.mdpi.com/article/10.3390/plants15203121/s1, Table S1: Information of commercial apple orchards surveyed in Northwestern Italy between 2023 and 2025; Table S2: Reference *Colletotrichum* strains included in the multilocus phylogenetic analyses and corresponding GenBank accession numbers for the loci used in this study; Table S3: Parsimony and Bayesian analyses characteristics in this study; Table S4: Nucleotide substitution models selected for each locus used in the multilocus phylogenetic analyses according to MrModeltest v.2.3; Table S5: In vitro sensitivity of apple-associated *Colletotrichum* isolates to fludioxonil, mefentrifluconazole, and pyraclostrobin, expressed as EC_50_ values (µg mL^−1^). “-”, EC_50_ value not determined; Figure S1: Visual reference scale used for the assessment of disease severity on apple leaves in pathogenicity assays. Scores correspond to the following symptomatic leaf-area classes: 0, no symptoms; 1, ≤5%; 2, 6–10%; 3, 11–25%; 4, 26–50%; and 5, 51–100%.
