# Supplementary material for: Diversity and Fungicide Sensitivity of Colletotrichum spp. Causing Apple Bitter Rot and Glomerella Leaf Spot in Northwestern Italy
Source: Plants (Basel). 2026 Oct 12;15(20):3121. doi: 10.3390/plants15203121 (PMC13709003; doi:10.3390/plants15203121)

Table S1. Information of commercial apple orchards surveyed in Northwestern Italy between 2023 and 2025.

Orchard n°	Location	Cultivar	Year(s) of isolation
**1**	Revello (CN), Italy	Gala	2023, 2024
**2**	Revello (CN), Italy	Gala	2023
**3**	Revello (CN), Italy	Gala	2023
**4**	Villaretto (CN), Italy	unknown	2024
**5**	Revello (CN), Italy	Gala	2024, 2025
**6**	Valgrana (CN), Italy	Golden Orange	2024
**7**	Scarnafigi (CN), Italy	SchniCo Red Gala Schinga®	2024
**8**	Chivasso (TO), Italy	Opal®	2024
**9**	Savigliano (CN), Italy	SchniCo Red Gala Schinga®	2024
**10**	Revello (CN), Italy	SchniCo Red Gala Schinga®	2024
**11**	Savigliano (CN), Italy	Crimson Snow®	2024
**12**	Savigliano (CN), Italy	SchniCo Red Gala Schinga®	2024
**13**	Lagnasco (CN), Italy	Crimson Snow®	2024
**14**	Scarnafigi (CN), Italy	Story® Inored	2024
**15**	Verzuolo (CN), Italy	Tessa® Fengapi	2024
**16**	Revello (CN), Italy	Golden Delicious	2024
**17**	Revello (CN), Italy	Simmons/Buckeye®	2024
**18**	Revello (CN), Italy	Story® Inored	2024
**19**	Scarnafigi (CN), Italy	Brookfield® Gala	2024
**20**	Revello (CN), Italy	Golden Delicious	2024
**21**	Revello (CN), Italy	Granny Smith	2024
**22**	Santhià (VC), Italy	Golden Orange	2024
**23**	Savigliano (CN), Italy	Story® Inored	2024
**24**	Revello (CN), Italy	SchniCo Red Gala Schinga®	2025
**25**	Scarnafigi (CN), Italy	Simmons/Buckeye®	2025
**26**	Scarnafigi (CN), Italy	Gala	2025
**27**	Castiglione Torinese (TO), Italy	Runsè	2025
**28**	Lagnasco (CN), Italy	ISAAQ	2025

Table S2. Reference *Colletotrichum* strains included in the multilocus phylogenetic analyses and corresponding GenBank accession numbers for the loci used in this study.

**Species complex**	**Species**	**Isolate**	**Genbank accession numbers**							
			gapdh	***chs-1***	***his3***	***act***	***tub2***	***cal***	***gs***	**ApMat**
C. acutatum SC	*Colletotrichum abscissum*	COAD 1877	KP843129	KP843132	KP843138	KP843141	KP843135	-	-	-
	Colletotrichum acerbum	CBS 128530	JQ948790	JQ949120	JQ949450	JQ949780	JQ950110	-	-	-
	Colletotrichum acutatum	CBS 112996	JQ948677	JQ005797	JQ005818	JQ005839	JQ005860	-	-	-
	Colletotrichum arboricola	CBS 144795	MH817950	OR645024	OR659702	MH817956	MH817962	-	-	-
	Colletotrichum australe	CBS 116478	JQ948786	JQ949116	JQ949446	JQ949776	JQ950106	-	-	-
	Colletotrichum bannaense	CGMCC 3.18887	MG242006	MG241996	-	MG242002	MG209660	-	-	-
	Colletotrichum brisbanense	CBS 292.67	JQ948621	JQ948952	JQ949282	JQ949612	JQ949942	-	-	-
	Colletotrichum cairnsense	BRIP 63642	KU923704	KU923710	KU923722	KU923716	KU923688	-	-	-
	Colletotrichum carthami	SAPA100011	-	-	-	-	AB696992	-	-	-
	Colletotrichum chrysanthemi	SAPA100010	-	-	-	-	AB696993	-	-	-
	Colletotrichum cosmi	CBS 853.73	JQ948604	JQ948935	JQ949265	JQ949595	JQ949925	-	-	-
	Colletotrichum costaricense	CBS 330.75	JQ948510	JQ948841	JQ949171	JQ949501	JQ949831	-	-	-
	Colletotrichum cuscutae	IMI 304802	JQ948525	JQ948856	JQ949186	JQ949516	JQ949846	-	-	-
	Colletotrichum eriobotryae	GLMC 1935	MF795423	MN191653	MN191658	MN191648	MF795428	-	-	-
	Colletotrichum filicis	CBS 101611	JQ948526	JQ948857	JQ949187	JQ949517	JQ949847	-	-	-
	Colletotrichum fioriniae	CBS 128517	JQ948622	JQ948953	JQ949283	JQ949613	JQ949943	-	-	-

Colletotrichum fioriniae (ex *C. orientale*)	CBS 128555	JQ948635	JQ948966	JQ949296	JQ949626	JQ949956	-	-	-
Colletotrichum fioriniae (ex *C. radermacherae*)	GZAAS 21-0300	OP737966	OP715771	-	OP715806	OP720862	-	-	-
Colletotrichum godetiae	CBS 127561	JQ948773	JQ949103	JQ949433	JQ949763	JQ950093	-	-	-
Colletotrichum godetiae	CBS 133.44	JQ948733	JQ949063	JQ949393	JQ949723	JQ950053	-	-	-
Colletotrichum godetiae	IMI 351248	JQ948764	JQ949094	JQ949424	JQ949754	JQ950084	-	-	-
Colletotrichum godetiae (ex *C. lauri*)	MFLUCC 17-0205	KY514344	KY514341	-	KY514338	KY514350	-	-	-
Colletotrichum guajavae	IMI 350839	JQ948600	JQ948931	JQ949261	JQ949591	JQ949921	-	-	-
Colletotrichum indonesiense	CBS 127551	JQ948618	JQ948949	JQ949279	JQ949609	JQ949939	-	-	-
Colletotrichum javanense	CBS 144963	MH846572	MH846573	MH846571	MH846575	MH846574	-	-	-
Colletotrichum johnstonii	CBS 128532	JQ948775	JQ949105	JQ949435	JQ949765	JQ950095	-	-	-
Colletotrichum kinghornii	CBS 198.35	JQ948785	JQ949115	JQ949445	JQ949775	JQ950105	-	-	-
Colletotrichum kniphofiae	CBS 143496	MH107998	MH107990	-	MH107975	MH108037	-	-	-
Colletotrichum laticiphilum	CBS 112989	JQ948619	JQ948950	JQ949280	JQ949610	JQ949940	-	-	-
Colletotrichum limetticola	CBS 114.14	JQ948523	JQ948854	JQ949184	JQ949514	JQ949844	-	-	-
Colletotrichum lupini	CBS 109225	JQ948485	JQ948816	JQ949146	JQ949476	JQ949806	-	-	-
Colletotrichum melonis	CBS 159.84	JQ948524	JQ948855	JQ949185	JQ949515	JQ949845	-	-	-
Colletotrichum miaoliense	NTUCC 20-001-1	MK908470	MK908522	-	MK908573	MK908624	-	-	-
Colletotrichum nymphaeae	CBS 129945	JQ948531	JQ948862	JQ949192	JQ949522	JQ949852	-	-	-
Colletotrichum nymphaeae	CBS 515.78	JQ948527	JQ948858	JQ949188	JQ949518	JQ949848	-	-	-

Colletotrichum nymphaeae	IMI 370491	JQ948534	JQ948865	JQ949195	JQ949525	JQ949855	-	-	-
Colletotrichum nymphaeae (ex *C. simulanticitri*)	YMF1.07302	OK513680	OK513577	-	OK513615	-	-	-	-
Colletotrichum paranaense	CBS 134729	KC205026	KC205043	KC205004	KC205077	KC205060	-	-	-
Colletotrichum paxtonii	IMI 165753	JQ948615	JQ948946	JQ949276	JQ949606	JQ949936	-	-	-
Colletotrichum pedunculi	BCRC FU31310	MN820684	MN820676	MN820692	MN820668	MN810342	-	-	-
Colletotrichum phormii	CBS 118194	JQ948777	JQ949107	JQ949437	JQ949767	JQ950097	-	-	-
Colletotrichum pyricola	CBS 128531	JQ948776	JQ949106	JQ949436	JQ949766	JQ950096	-	-	-
Colletotrichum rhombiforme	CBS 129953	JQ948788	JQ949118	JQ949448	JQ949778	JQ950108	-	-	-
Colletotrichum roseum	CBS 145754	MK903603	OR645057	OR659735	MK903604	-	-	-	-
Colletotrichum salicis	CBS 607.94	JQ948791	JQ949121	JQ949451	JQ949781	JQ950111	-	-	-
Colletotrichum schimae	NN046984	MZ664105	MZ799347	MZ673905	MZ664183	MZ674003	-	-	-
Colletotrichum scovillei	CBS 126529	JQ948597	JQ948928	JQ949258	JQ949588	JQ949918	-	-	-
Colletotrichum simmondsii	CBS 122122	JQ948606	JQ948937	JQ949267	JQ949597	JQ949927	-	-	-
Colletotrichum sloanei	IMI 364297	JQ948617	JQ948948	JQ949278	JQ949608	JQ949938	-	-	-
Colletotrichum subsalicis	LC13863	MZ664104	MZ799346	MZ673836	MZ664128	MZ673953	-	-	-
Colletotrichum tamarilloi	CBS 129814	JQ948514	JQ948845	JQ949175	JQ949505	JQ949835	-	-	-
Colletotrichum walleri	CBS 125472	JQ948605	JQ948936	JQ949266	JQ949596	JQ949926	-	-	-
Colletotrichum wanningense	CGMCC 3.18936	MG830318	MG830302	-	MG830270	MG830286	-	-	-
Colletotrichum wanningense (ex *C. australisinense*)	CGMCC 3.18886	MG241962	MG241981	-	MG241947	MG209645	-	-	-

C. gloeosporioides SC	*Colletotrichum aenigma*	ICMP 18608	JX010044	JX009774	-	JX009443	JX010389	JX009683	JX010078	KM360143
	Colletotrichum aeschynomenes	ICMP 17673	JX009930	JX009799	-	JX009483	JX010392	JX009721	JX010081	KM360145
	Colletotrichum alatae	ICMP 17919	JX009990	JX009837	-	JX009471	JX010383	JX009738	JX010065	KC888932
	Colletotrichum alienum	CBS 133930	KC297000	KC296982	KC297038	KC296938	KC297096	KC296957	KC297023	-
	Colletotrichum alienum	ICMP 12071	JX010028	JX009882	-	JX009572	JX010411	JX009654	JX010101	KM360144
	Colletotrichum analogum	YMF1.06943	OK513663	OK513559	-	OK513599	OK513629	-	-	-
	Colletotrichum aotearoa	ICMP 18537	JX010005	JX009853	-	JX009564	JX010420	JX009611	JX010113	KC888930
	Colletotrichum arecicola	CGMCC 3.19667	MK935455	MK935541	-	MK935374	MK935498	-	-	MK935413
	Colletotrichum artocarpicola	MFLUCC 18-1167	MN435568	MN435569	-	MN435570	MN435567	-	-	-
	Colletotrichum asianum	ICMP 18580	JX010053	JX009867	-	JX009584	JX010406	FJ917506	JX010096	FR718814
	Colletotrichum australianum	VPRI 43075	MG572127	MW091987	-	MN442109	MG572149	-	MG572160	MG572171
	Colletotrichum camelliae	CGMCC 3.14925	KJ954782	-	-	KJ954363	KJ955230	KJ954634	KJ954932	KJ954497
	Colletotrichum cangyuanense	YMF1.05000	OK513666	OK513562	-	OK513602	OK513632	-	-	-
	Colletotrichum castaneae	HGUP 22097	OP737974	OP715779	-	OP715813	-	-	-	-
	Colletotrichum changpingense	MFLUCC 15-0022	KP852469	KP852449	-	KP683093	KP852490	-	-	-
	Colletotrichum chiangmaiense	MFLU 20–0604	MW548592	MW623653	-	MW655578	-	-	-	-
	Colletotrichum chrysophilum	CMM4268	KX094183	KX094083	-	KX093982	KX094285	KX094063	KX094204	KX094325
	Colletotrichum chrysophilum	CPO 27.222	MK507867	MK507870	-	MK507873	MK507879	-	MK507876	MK507882
	Colletotrichum chrysophilum	LM1270	OQ745587	-	-	-	OQ818242	OQ831800	OQ791454	OQ791443
	Colletotrichum cigarro	ICMP 18539	JX009966	JX009800	-	JX009523	JX010434	JX009635	JX010132	-

Colletotrichum clidemiae	ICMP 18658	JX009989	JX009877	-	JX009537	JX010438	JX009645	JX010129	KC888929
Colletotrichum cobbittiense	BRIP 66219	MH094133	MH094135	-	MH094134	MH094137	-	-	-
Colletotrichum conoides	CGMCC 3.17615	KP890162	KP890156	-	KP890144	KP890174	KP890150	-	-
Colletotrichum cordylinicola	ICMP 18579	JX009975	JX009864	-	HM470235	JX010440	HM470238	JX010122	JQ899274
Colletotrichum dracaenigenum	MFLUCC 19–0430	MT215577	MT215575	-	MT313686	-	-	-	-
Colletotrichum endophyticum	MFLUCC 13-0418	KC832854	-	MZ673839	KF306258	MZ673954	KC810018	-	-
Colletotrichum fici-septicae	MFLU 19-2770	MW183774	MW177701	-	MW151585	-	-	-	-
Colletotrichum fructicola	ICMP 18581	JX010033	JX009866	-	FJ907426	JX010405	FJ917508	JX010095	JQ807838
Colletotrichum fructicola (ex *C. mengyinense*)	SAUCC200702	MW846240	MW883686	-	MW883695	MW888970	MW922538	MW888961	-
Colletotrichum fructivorum	CBS 133125	MZ664047	MZ799259	-	MZ664126	JX145196	-	-	JX145300
Colletotrichum gardeniae	HGUP 22032	OP737963	OP715766	-	OP715801	OP720858	-	-	-
Colletotrichum gloeosporioides	CBS 112999	JQ005239	JQ005326	-	JQ005500	JQ005587	JQ005673	JX010085	JQ807843
Colletotrichum gloeosporioides (ex *C. citrulli*)	CAASZT52	OL456685	OL901153	ON479082	OL449283	OL456644	-	-	-
Colletotrichum gloeosporioides (ex *C. dimorphum*)	YMF1.07303	OK513669	OK513565	-	OK513605	OK513635	-	-	-
Colletotrichum gloeosporioides (ex *C. nanhuaense*)	YMF1.04993	OK513673	OK513569	-	OK513609	OK513639	-	-	-
Colletotrichum gracile	YMF1.06939	OK513671	OK513567	-	OK513607	OK513637	-	-	-
Colletotrichum grevilleae	CBS 132879	KC297010	KC296987	KC297056	KC296941	KC297102	KC296963	KC297033	-
Colletotrichum grossum	CGMCC3.17614	KP890159	KP890153	-	KP890141	KP890171	KP890147	-	-
Colletotrichum hebeiense	MFLUCC 13-0726	KF377495	KF289008	-	KF377532	KF288975	-	-	KF377562

Colletotrichum hederiicola	MFLU 15-0689	-	MN635794	-	MN635795	-	-	-	-
Colletotrichum helleniense	CBS 142418	KY856270	KY856186	-	KY856019	KY856528	KY856099	-	-
Colletotrichum henanense	CGMCC 3.17354	KJ954810	MZ799256	-	KM023257	KJ955257	KJ954662	KJ954960	KJ954524
Colletotrichum horii	ICMP 10492	GQ329681	JX009752	-	JX009438	JX010450	JX009604	JX010137	JQ807840
Colletotrichum hystricis	CBS 142411	KY856274	KY856190	-	KY856023	KY856532	KY856103	-	-
Colletotrichum hystricis	CPC 28154=CBS 142412	KY856275	KY856191	KY856366	KY856024	KY856533	KY856104	-	-
Colletotrichum jiangxiense	CGMCC 3.17363	KJ954902	-	-	KJ954471	KJ955348	KJ954752	KJ955051	KJ954607
Colletotrichum kahawae	ICMP 17816	JX010012	JX009813	-	JX009452	JX010444	JX009642	JX010130	JQ894579
Colletotrichum kunmingense	HGUP 22049	OP737965	OP715769	-	OP715804	OP720861	-	-	-
Colletotrichum ledongense	CGMCC 3.18888	MG242017	MG242019	-	MG242015	MG242011	MG242013	MG242021	-
Colletotrichum ligustri	HGUP 22051	OP737968	OP715773	-	OP740216	OP720864	-	-	-
Colletotrichum makassarense	CBS 143664	MH728820	MH805850	-	MH781480	MH846563	-	MH748264	MH728831
Colletotrichum menglaense	YMF 1.04960	MH023507	MH023508	-	MH023506	-	-	-	-
Colletotrichum noveboracense	AFKH109	MN640567	-	-	MN640565	MN640569	MN640566	MN640568	MN640564
Colletotrichum nullisetosum	YMF1.06946	OK513675	OK513571	-	OK513611	OK513641	-	-	-
Colletotrichum nupharicola	ICMP 18187	JX009972	JX009835	-	JX009437	JX010398	JX009663	JX010088	JX145319
Colletotrichum oblongisporum	YMF1.06938	OK513677	OK513573	-	-	OK513643	-	-	-
Colletotrichum pandanicola	MFLUCC 17-0571	MG646934	MG646931	-	MG646938	MG646926	-	-	-
Colletotrichum parvisporum	YMF1.06942	OK513679	OK513575	-	OK513613	OK513645	-	-	-

Colletotrichum perseae	CBS 141365	KX620242	MZ799260	-	KX620145	KX620341	KX620206	KX620275	KX620177
Colletotrichum proteae	CBS 132882	KC297009	KC296986	-	KC296940	KC297101	KC296960	-	-
Colletotrichum pseudotheobromicola	MFLUCC 18–1602	MH853675	MH853678	-	MH853681	MH853684	-	-	-
Colletotrichum psidii	ICMP 19120	JX009967	JX009901	-	JX009515	JX010443	JX009743	JX010133	KC888931
Colletotrichum queenslandicum	ICMP 1778	JX009934	JX009899	-	JX009447	JX010414	JX009691	JX010104	KC888928
Colletotrichum rhexiae	CBS 133134	MZ664046	MZ799258	MZ673834	MZ664127	JX145179	-	-	JX145290
Colletotrichum salsolae	ICMP 19051	JX009916	JX009863	-	JX009562	JX010403	JX009696	JX010093	KC888925
Colletotrichum siamense	ICMP 18578	JX009924	JX009865	-	FJ907423	JX010404	FJ917505	JX010094	JQ899289
Colletotrichum subhenanense	YMF1.06865	OK513684	OK513581	-	OK513618	OK513647	-	-	-
Colletotrichum syzygiicola	MFLUCC 10-0624	KF242156	-	-	KF157801	KF254880	KF254859	KF242125	-
Colletotrichum tainanense	CBS 143666	MH728823	MH805845	-	MH781475	MH846558	-	MH748259	MH728836
Colletotrichum temperatum	CBS 133122	MZ664045	MZ799254	MZ673833	MZ664125	JX145211	-	-	JX145298
Colletotrichum thasutense	MFLU 22-0206	OP831282	OP831281	-	OP831280	OP831283	-	-	-
Colletotrichum theobromicola	ICMP 18649	JX010006	JX009869	-	JX009444	JX010447	JX009591	JX010139	KC790726
Colletotrichum ti	ICMP 4832	JX009952	JX009898	-	JX009520	JX010442	JX009649	JX010123	KM360146
Colletotrichum tomentosae	ZHKUCC 21-0103	OL855850	OL855860	-	OL855870	OL855887	-	-	-
Colletotrichum tropicale	ICMP 18653	JX010007	JX009870	-	JX009489	JX010407	JX009719	JX010097	KC790728
Colletotrichum viniferum	GZAAS 5.08601	JN412798	-	-	JN412795	JN412813	JQ309639	JN412787	-
Colletotrichum wuxiense	CGMCC 3.17894	KU252045	KU251939	-	KU251672	KU252200	KU251833	KU252101	KU251722

	Colletotrichum xanthorrhoeae	ICMP 17903	JX009927	JX009823	-	JX009478	JX010448	JX009653	JX010138	KC790689
	Colletotrichum xishuangbannaense	MFLUCC 19-0107	MW537586	MW660832	-	MW652294	-	-	-	-
	Colletotrichum yuanjiangense	YMF1.04996	OK513686	OK513583	-	OK513620	OK513649	-	-	-
	Colletotrichum yulongense	CFCC 50819	MK108992	MK108991	-	MK108989	MK108993	MK108990	MK108995	-
outgroup	*Colletotrichum orchidophilum*	CBS 632.80	JQ948481	JQ948812	JQ949142	JQ949472	JQ949802	-	-	-
	Colletotrichum pyrifoliae	CGMCC 3.18902	MG747996	MG747914	-	MG747768	MG748158	-	-	-

Table S3. Parsimony and Bayesian analyses characteristics in this study.

		***Colletotrichum gloeosporioides* SC**	***Colletotrichum acutatum* SC**
	Locus(i)	*gapdh, chs-1, act, tub2, cal, gs*, ApMat	*gapdh, chs-1, his3, act, tub2*
**Parsimony analysis**	Total sites	3,570	1,629
	Constant sites	1745	1038
	Variable sites	1014	241
	Parsimony Informative sites	775	326
	Tree length	3,142	1,126
	Consistency index	0.739	0.663
	Retention index	0.879	0.870
	Rescaled consistence index	0.649	0.577
**Bayesian analysis**	Unique site patterns of *gapdh*	165	161
	Unique site patterns of *chs-1*	85	72
	Unique site patterns of *his3*	-	103
	Unique site patterns of act	120	95
	Unique site patterns of *tub2*	269	157
	Unique site patterns of *cal*	216	-
	Unique site patterns of *gs*	339	-
	Unique site patterns of ApMat	473	-
	Generation ran	23,205,000	975000
	Generated trees	46412	1952
	Sampled trees	34810	1464

Table S4. Nucleotide substitution models selected for each locus used in the multilocus phylogenetic analyses according to MrModeltest v.2.3 .

Species complex	Locus	Evolutionary model	State frequencies
C. gloeosporioides SC	*gapdh*	HKY+G	Dirichlet
	chs-1	K80+G	Equal
	act	K80+G	Equal
	tub2	GTR+G	Dirichlet
	cal	GTR+G	Equal
	gs	HKY+G	Dirichlet
	ApMat	HKY+G	Equal
C. acutatum SC	*gapdh*	K80+G	Equal
	chs-1	GTR+I+G	Equal
	his3	GTR+G	Dirichlet
	act	HKY+G	Dirichlet
	tub2	HKY+G	Dirichlet

Table S5. *In vitro* sensitivity of apple-associated *Colletotrichum* isolates to fludioxonil, mefentrifluconazole, and pyraclostrobin, expressed as EC_{50} values (μg mL^{-1}). "-", EC_{50} value not determined.

Code	Species	Species complex	Host and Cultivar	Organ	Orchard n°	Year of isolation	EC_{50} (μg mL^{-1})		
							Fludioxonil	Mefentrifluconazole	Pyraclostrobin
CVG 2240	*Colletotrichum chrysophilum*	*C. gloeosporioides* SC	*Malus* × *domestica* cv Gala	fruit	1	2023	0,0099	0,0931	-
CVG 2241	*Colletotrichum chrysophilum*	*C. gloeosporioides* SC	*Malus* × *domestica* cv Gala	fruit	1	2023	0,0074	0,0931	0,0024
CVG 2242	*Colletotrichum chrysophilum*	*C. gloeosporioides* SC	*Malus* × *domestica* cv Gala	fruit	1	2023	0,0075	0,0878	-
CVG 2243	*Colletotrichum gloeosporioides* SC	*C. gloeosporioides* SC	*Malus* × *domestica* cv Gala	fruit	1	2023	0,0074	0,0913	0,0040
CVG 2244	*Colletotrichum gloeosporioides* SC	*C. gloeosporioides* SC	*Malus* × *domestica* cv Gala	fruit	1	2023	0,0075	0,0931	-
CVG 2245	*Colletotrichum gloeosporioides* SC	*C. gloeosporioides* SC	*Malus* × *domestica* cv Gala	fruit	1	2023	0,0083	0,0878	-
CVG 2246	*Colletotrichum gloeosporioides* SC	*C. gloeosporioides* SC	*Malus* × *domestica* cv Gala	fruit	1	2023	0,0086	0,1272	0,0034
CVG 2247	*Colletotrichum chrysophilum*	*C. gloeosporioides* SC	*Malus* × *domestica* cv Gala	leaf	1	2023	0,0088	0,0782	0,6216
CVG 2248	*Colletotrichum chrysophilum*	*C. gloeosporioides* SC	*Malus* × *domestica* cv Gala	leaf	1	2023	0,0118	0,0845	0,8803
CVG 2249	*Colletotrichum chrysophilum*	*C. gloeosporioides* SC	*Malus* × *domestica* cv Gala	leaf	1	2023	0,0105	0,0350	1,4075
CVG 2250	*Colletotrichum gloeosporioides* SC	*C. gloeosporioides* SC	*Malus* × *domestica* cv Gala	leaf	1	2023	0,0078	0,0697	0,7257
CVG 2251	*Colletotrichum gloeosporioides* SC	*C. gloeosporioides* SC	*Malus* × *domestica* cv Gala	leaf	1	2023	0,0083	0,0657	0,8634
CVG 2252	*Colletotrichum gloeosporioides* SC	*C. gloeosporioides* SC	*Malus* × *domestica* cv Gala	leaf	1	2023	0,0085	0,0652	0,5641
CVG 2253	*Colletotrichum chrysophilum*	*C. gloeosporioides* SC	*Malus* × *domestica* cv Gala	leaf	2	2023	0,0080	0,0391	1,7225

CVG 2254	*Colletotrichum chrysophilum*	*C. gloeosporioides* SC	*Malus × domestica* cv Gala	leaf	2	2023	0,0293	0,0767	0,0005
CVG 2255	*Colletotrichum chrysophilum*	*C. gloeosporioides* SC	*Malus × domestica* cv Gala	leaf	2	2023	0,0070	0,0949	0,3801
CVG 2256	*Colletotrichum gloeosporioides* SC	*C. gloeosporioides* SC	*Malus × domestica* cv Gala	leaf	2	2023	0,0075	0,0861	0,8307
CVG 2257	*Colletotrichum gloeosporioides* SC	*C. gloeosporioides* SC	*Malus × domestica* cv Gala	leaf	2	2023	0,0075	0,0986	0,0045
CVG 2258	*Colletotrichum gloeosporioides* SC	*C. gloeosporioides* SC	*Malus × domestica* cv Gala	leaf	2	2023	0,0077	0,0845	0,0022
CVG 2259	*Colletotrichum chrysophilum*	*C. gloeosporioides* SC	*Malus × domestica* cv Gala	fruit	3	2023	0,0081	0,0861	0,0032
CVG 2260	*Colletotrichum chrysophilum*	*C. gloeosporioides* SC	*Malus × domestica* cv Gala	fruit	3	2023	0,0186	0,0657	0,0027
CVG 2261	*Colletotrichum chrysophilum*	*C. gloeosporioides* SC	*Malus × domestica* cv Gala	fruit	3	2023	0,0067	0,0895	-
CVG 2262	*Colletotrichum gloeosporioides* SC	*C. gloeosporioides* SC	*Malus × domestica* cv Gala	fruit	3	2023	0,0073	0,0356	0,0018
CVG 2263	*Colletotrichum gloeosporioides* SC	*C. gloeosporioides* SC	*Malus × domestica* cv Gala	fruit	3	2023	0,0086	0,0422	0,0041
CVG 2264	*Colletotrichum gloeosporioides* SC	*C. gloeosporioides* SC	*Malus × domestica* cv Gala	fruit	3	2023	0,0067	0,0329	-
CVG 2265	*Colletotrichum chrysophilum*	*C. gloeosporioides* SC	*Malus × domestica* cv Gala	leaf	3	2023	0,0077	0,0797	-
CVG 2266	*Colletotrichum chrysophilum*	*C. gloeosporioides* SC	*Malus × domestica* cv Gala	leaf	3	2023	0,0090	0,0439	0,0016
CVG 2267	*Colletotrichum chrysophilum*	*C. gloeosporioides* SC	*Malus × domestica* cv Gala	leaf	3	2023	0,0077	0,0376	-
CVG 2268	*Colletotrichum gloeosporioides* SC	*C. gloeosporioides* SC	*Malus × domestica* cv Gala	leaf	3	2023	0,0097	0,0474	-
CVG 2269	*Colletotrichum gloeosporioides* SC	*C. gloeosporioides* SC	*Malus × domestica* cv Gala	leaf	3	2023	0,0080	0,0829	0,0017
CVG 2270	*Colletotrichum gloeosporioides* SC	*C. gloeosporioides* SC	*Malus × domestica* cv Gala	leaf	3	2023	0,0095	0,0767	0,0021

CVG 2454	*Colletotrichum fioriniae*	*C. acutatum* SC	*Malus* × *domestica* cv unknown	leaf	4	2024	0,0091	0,0448	0,0003
CVG 2455	*Colletotrichum acutatum SC* (*C. fioriniae* clade)	*C. acutatum* SC	*Malus* × *domestica* cv unknown	fruit	4	2024	0,0075	0,0369	0,0010
CVG 2456	*Colletotrichum acutatum SC* (*C. fioriniae* clade)	*C. acutatum* SC	*Malus* × *domestica* cv unknown	fruit	4	2024	0,0065	-	0,0003
CVG 2457	*Colletotrichum fioriniae*	*C. acutatum* SC	*Malus* × *domestica* cv unknown	fruit	4	2024	0,0075	0,0369	0,0003
CVG 2458	*Colletotrichum acutatum SC* (*C. fioriniae* clade)	*C. acutatum* SC	*Malus* × *domestica* cv unknown	fruit	4	2024	0,0074	0,0362	0,0004
CVG 2459	*Colletotrichum acutatum SC* (*C. fioriniae* clade)	*C. acutatum* SC	*Malus* × *domestica* cv unknown	fruit	4	2024	0,0091	0,0448	0,0003
CVG 2460	*Colletotrichum acutatum SC* (*C. fioriniae* clade)	*C. acutatum* SC	*Malus* × *domestica* cv unknown	fruit	4	2024	0,0078	0,0384	0,0003
CVG 2464	*Colletotrichum gloeosporioides*	*C. gloeosporioides* SC	*Malus* × *domestica* cv Gala	leaf	5	2024	0,0093	0,0829	-
CVG 2465	*Colletotrichum gloeosporioides*	*C. gloeosporioides* SC	*Malus* × *domestica* cv Gala	leaf	5	2024	0,0074	0,0362	0,0025
CVG 2466	*Colletotrichum godetiae*	*C. acutatum* SC	*Malus* × *domestica* cv Golden orange	fruit	6	2024	0,0047	0,0560	0,0002
CVG 2467	*Colletotrichum chrysophilum*	*C. gloeosporioides* SC	*Malus* × *domestica* cv SchniCo Red Gala Schinga®	fruit	7	2024	0,0097	0,0697	0,6717
CVG 2470	*Colletotrichum nymphaeae*	*C. acutatum* SC	*Malus* × *domestica* cv Opal®	fruit	8	2024	0,0055	0,0620	0,0005
CVG 2471	*Colletotrichum acutatum SC* (*C. nymphaeae* clade)	*C. acutatum* SC	*Malus* × *domestica* cv Opal®	fruit	8	2024	0,0075	0,0531	0,0003
CVG 2472	*Colletotrichum acutatum SC* (*C. nymphaeae* clade)	*C. acutatum* SC	*Malus* × *domestica* cv Opal®	fruit	8	2024	0,0080	0,0585	0,0005
CVG 2473	*Colletotrichum chrysophilum*	*Colletotrichum gloeosporioides* SC	*Malus* × *domestica* cv Opal®	fruit	8	2024	0,0078	0,0384	-
CVG 2474	*Colletotrichum gloeosporioides* SC	*C. gloeosporioides* SC	*Malus* × *domestica* cv Opal®	fruit	8	2024	0,0090	0,0767	-
CVG 2475	*Colletotrichum fioriniae*	*C. acutatum* SC	*Malus* × *domestica* cv Opal®	fruit	8	2024	0,0074	0,0362	-

CVG 2476	*Colletotrichum acutatum SC* (*C. fioriniae* clade)	*C. acutatum* SC	*Malus × domestica* cv Opal®	fruit	8	2024	0,0070	0,0342	0,0013
CVG 2478	*Colletotrichum gloeosporioides* SC	*C. gloeosporioides* SC	*Malus × domestica* cv SchniCo Red Gala Schinga®	leaf	9	2024	0,0080	0,1130	0,4920
CVG 2481	*Colletotrichum gloeosporioides* SC	*C. gloeosporioides* SC	*Malus × domestica* cv SchniCo Red Gala Schinga®	leaf	9	2024	0,0095	0,0861	0,4371
CVG 2484	*Colletotrichum gloeosporioides* SC	*C. gloeosporioides* SC	*Malus × domestica* cv SchniCo Red Gala Schinga®	leaf	10	2024	0,0175	0,0878	-
CVG 2485	*Colletotrichum gloeosporioides* SC	*C. gloeosporioides* SC	*Malus × domestica* cv SchniCo Red Gala Schinga®	leaf	10	2024	0,0113	0,0620	0,5321
CVG 2486	*Colletotrichum gloeosporioides* SC	*C. gloeosporioides* SC	*Malus × domestica* cv SchniCo Red Gala Schinga®	leaf	10	2024	0,0080	0,0632	0,0019
CVG 2487	*Colletotrichum acutatum SC* (*C. fioriniae* clade)	*C. acutatum* SC	*Malus × domestica* cv SchniCo Red Gala Schinga®	leaf	10	2024	0,0073	0,0355	0,0003
CVG 2488	*Colletotrichum fioriniae*	*C. acutatum* SC	*Malus × domestica* cv SchniCo Red Gala Schinga®	leaf	10	2024	0,0075	0,0369	0,0006
CVG 2489	*Colletotrichum acutatum SC* (*C. fioriniae* clade)	*C. acutatum* SC	*Malus × domestica* cv SchniCo Red Gala Schinga®	leaf	10	2024	0,0077	0,0376	0,0005
CVG 2490	*Colletotrichum gloeosporioides* SC	*C. gloeosporioides* SC	*Malus × domestica* cv Crimson Snow®	leaf	11	2024	0,0088	0,0431	1,4946
CVG 2492	*Colletotrichum chrysophilum*	*C. gloeosporioides* SC	*Malus × domestica* cv Crimson Snow®	leaf	11	2024	0,0088	0,0431	-
CVG 2493	*Colletotrichum gloeosporioides* SC	*C. gloeosporioides* SC	*Malus × domestica* cv Crimson Snow®	fruit	11	2024	0,0091	0,0448	0,0025
CVG 2496	*Colletotrichum gloeosporioides* SC	*C. gloeosporioides* SC	*Malus × domestica* cv Crimson Snow®	fruit	11	2024	0,0070	0,1297	0,0030
CVG 2497	*Colletotrichum gloeosporioides* SC	*C. gloeosporioides* SC	*Malus × domestica* cv Crimson Snow®	fruit	11	2024	0,0103	0,0503	-
CVG 2498	*Colletotrichum chrysophilum*	*C. gloeosporioides* SC	*Malus × domestica* cv SchniCo Red Gala Schinga®	leaf	12	2024	0,0086	0,0422	-
CVG 2500	*Colletotrichum gloeosporioides* SC	*C. gloeosporioides* SC	*Malus × domestica* cv SchniCo Red Gala Schinga®	leaf	12	2024	0,0085	0,0414	0,0027
CVG 2501	*Colletotrichum gloeosporioides* SC	*C. gloeosporioides* SC	*Malus × domestica* cv SchniCo Red Gala Schinga®	fruit	12	2024	0,0103	0,0503	0,0022

CVG 2502	*Colletotrichum gloeosporioides* SC	*C. gloeosporioides* SC	*Malus* × *domestica* cv SchniCo Red Gala Schinga®	fruit	12	2024	0,0146	0,0716	-
CVG 2503	*Colletotrichum chrysophilum*	*C. gloeosporioides* SC	*Malus* × *domestica* cv SchniCo Red Gala Schinga®	fruit	12	2024	0,0097	0,0474	0,0021
CVG 2505	*Colletotrichum gloeosporioides* SC	*C. gloeosporioides* SC	*Malus* × *domestica* cv Crimson Snow®	fruit	13	2024	0,0081	0,0399	-
CVG 2506	*Colletotrichum gloeosporioides* SC	*C. gloeosporioides* SC	*Malus* × *domestica* cv Crimson Snow®	fruit	13	2024	0,0093	0,0456	0,0020
CVG 2508	*Colletotrichum chrysophilum*	*C. gloeosporioides* SC	Malus × domestica cv Story® Inored	fruit	14	2024	0,0093	0,0456	-
CVG 2509	*Colletotrichum gloeosporioides* SC	*C. gloeosporioides* SC	Malus × domestica cv Story® Inored	fruit	14	2024	0,0034	0,0165	0,0004
CVG 2510	*Colletotrichum gloeosporioides* SC	*C. gloeosporioides* SC	*Malus* × *domestica* cv Tessa® Fengapi	fruit	15	2024	0,0078	0,0829	0,0026
CVG 2513	*Colletotrichum gloeosporioides* SC	*C. gloeosporioides* SC	*Malus* × *domestica* cv Tessa® Fengapi	fruit	15	2024	0,0109	0,0533	0,8307
CVG 2514	*Colletotrichum gloeosporioides* SC	*C. gloeosporioides* SC	*Malus* × *domestica* cv Tessa® Fengapi	fruit	15	2024	0,0099	0,0484	-
CVG 2515	*Colletotrichum chrysophilum*	*C. gloeosporioides* SC	*Malus* × *domestica* cv Tessa® Fengapi	fruit	15	2024	0,0090	0,0439	0,0034
CVG 2516	*Colletotrichum gloeosporioides* SC	*C. gloeosporioides* SC	*Malus* × *domestica* cv Tessa® Fengapi	fruit	15	2024	0,0083	0,0861	0,6097
CVG 2517	*Colletotrichum fioriniae*	*C. acutatum* SC	Malus × domestica cv Golden Delicious	leaf	16	2024	0,0101	0,0493	0,0008
CVG 2518	*Colletotrichum acutatum SC* (*C. fioriniae* clade)	*C. acutatum* SC	*Malus* × *domestica* cv Golden Delicious	leaf	16	2024	0,0066	0,0895	0,0005
CVG 2519	*Colletotrichum acutatum SC* (*C. fioriniae* clade)	*C. acutatum* SC	*Malus* × *domestica* cv Golden Delicious	leaf	16	2024	0,0125	0,0611	0,0005
CVG 2520	*Colletotrichum acutatum SC* (*C. fioriniae* clade)	*C. acutatum* SC	*Malus* × *domestica* cv Golden Delicious	leaf	16	2024	0,0077	0,0376	0,0005
CVG 2521	*Colletotrichum acutatum SC* (*C. fioriniae* clade)	*C. acutatum* SC	*Malus* × *domestica* cv Golden Delicious	leaf	16	2024	0,0074	0,0585	0,0005
CCI 202	*Colletotrichum chrysophilum*	*C. gloeosporioides* SC	*Malus* x *domestica* cv Simmons/Buckeye®	leaf	17	2024	0,0063	0,1433	1,4359

CCI 203	*Colletotrichum gloeosporioides* SC	*C. gloeosporioides* SC	*Malus* x *domestica* cv Simmons/Buckeye®	leaf	17	2024	0,0266	0,0931	-
CCI 204	*Colletotrichum gloeosporioides* SC	*C. gloeosporioides* SC	*Malus* x *domestica* cv Simmons/Buckeye®	leaf	17	2024	0,0030	0,0986	-
CCI 205	*Colletotrichum chrysophilum*	*C. gloeosporioides* SC	*Malus* x *domestica* cv Story® Inored	frui t	18	2024	0,0132	0,0986	-
CCI 206	*Colletotrichum gloeosporioides* SC	*C. gloeosporioides* SC	*Malus* x *domestica* cv Story® Inored	frui t	18	2024	0,0055	0,0738	-
CCI 207	*Colletotrichum gloeosporioides* SC	*C. gloeosporioides* SC	*Malus* x *domestica* cv Story® Inored	frui t	18	2024	0,0071	0,0348	-
CCI 208	*Colletotrichum nymphaeae*	*C. acutatum* SC	*Malus* x *domestica* cv Brookfield® Gala	leaf	19	2024	0,0083	0,0406	0,0062
CCI 209	*Colletotrichum acutatum* SC (*C. nymphaeae* clade)	*C. acutatum* SC	*Malus* x *domestica* cv Brookfield® Gala	leaf	19	2024	0,0103	0,0503	0,0023
CCI 210	*Colletotrichum acutatum* SC (*C. nymphaeae* clade)	*C. acutatum* SC	*Malus* x *domestica* cv Brookfield® Gala	leaf	19	2024	0,0083	0,0406	0,0005
CCI 211	*Colletotrichum chrysophilum*	*C. gloeosporioides* SC	*Malus* × *domestica* cv Gala	frui t	1	2024	0,0205	0,0845	-
CCI 212	*Colletotrichum gloeosporioides* SC	*C. gloeosporioides* SC	*Malus* × *domestica* cv Gala	frui t	1	2024	-	0,0913	-
CCI 213	*Colletotrichum chrysophilum*	*C. gloeosporioides* SC	*Malus* × *domestica* cv Gala	leaf	1	2024	0,0061	0,0845	-
CCI 214	*Colletotrichum gloeosporioides* SC	*C. gloeosporioides* SC	*Malus* × *domestica* cv Gala	leaf	1	2024	0,0095	0,0465	-
CCI 215	*Colletotrichum chrysophilum*	*C. gloeosporioides* SC	*Malus* × *domestica* cv Golden Delicious	frui t	20	2024	0,0085	0,0414	-
CCI 216	*Colletotrichum gloeosporioides* SC	*C. gloeosporioides* SC	*Malus* × *domestica* cv Golden Delicious	frui t	20	2024	0,0088	0,0431	-
CCI 217	*Colletotrichum gloeosporioides* SC	*C. gloeosporioides* SC	*Malus* × *domestica* cv Golden Delicious	frui t	20	2024	0,0023	0,0738	-
CCI 218	*Colletotrichum gloeosporioides* SC	*C. gloeosporioides* SC	*Malus* × *domestica* cv Golden Delicious	frui t	20	2024	0,0081	0,0931	-
CVG 2548	*Colletotrichum chrysophilum*	*C. gloeosporioides* SC	*Malus* × *domestica* cv SchniCo Red Gala Schinga®	frui t	9	2024	0,0255	0,1109	-

CVG 2549	*Colletotrichum gloeosporioides* SC	*C. gloeosporioides* SC	*Malus × domestica* cv SchniCo Red Gala Schinga®	fruit	9	2024	0,0045	0,0574	-
CVG 2550	*Colletotrichum gloeosporioides* SC	*C. gloeosporioides* SC	*Malus × domestica* cv SchniCo Red Gala Schinga®	fruit	9	2024	0,0063	0,0931	-
CVG 2551	*Colletotrichum chrysophilum*	*C. gloeosporioides* SC	*Malus × domestica* cv SchniCo Red Gala Schinga®	fruit	10	2024	0,0107	0,0967	0,2625
CVG 2552	*Colletotrichum gloeosporioides* SC	*C. gloeosporioides* SC	*Malus × domestica* cv SchniCo Red Gala Schinga®	fruit	10	2024	0,0061	0,1025	-
CVG 2553	*Colletotrichum gloeosporioides* SC	*C. gloeosporioides* SC	*Malus × domestica* cv SchniCo Red Gala Schinga®	fruit	10	2024	0,0071	0,0697	-
CVG 2554	*Colletotrichum chrysophilum*	*C. gloeosporioides* SC	*Malus × domestica* cv Granny smith	fruit	21	2024	0,0090	0,0753	-
CVG 2555	*Colletotrichum gloeosporioides* SC	*C. gloeosporioides* SC	*Malus × domestica* cv Granny smith	fruit	21	2024	0,0073	0,0782	-
CVG 2556	*Colletotrichum gloeosporioides* SC	*C. gloeosporioides* SC	*Malus × domestica* cv Granny smith	fruit	21	2024	0,0039	0,0657	-
CVG 2557	*Colletotrichum nymphaeae*	*C. acutatum* SC	*Malus × domestica* cv Golden orange	fruit	22	2024	0,0039	0,0895	0,0006
CVG 2558	*Colletotrichum acutatum* SC (*C. nymphaeae* clade)	*C. acutatum* SC	*Malus × domestica* cv Golden orange	fruit	22	2024	0,0018	0,0463	0,0003
CVG 2559	*Colletotrichum acutatum* SC (*C. nymphaeae* clade)	*C. acutatum* SC	*Malus × domestica* cv Golden orange	fruit	22	2024	0,0016	0,0845	0,0008
CVG 2560	*Colletotrichum acutatum* SC (*C. nymphaeae* clade)	*C. acutatum* SC	*Malus × domestica* cv Golden orange	fruit	22	2024	0,0020	0,0372	0,0002
CVG 2561	*Colletotrichum acutatum* SC (*C. nymphaeae* clade)	*C. acutatum* SC	*Malus × domestica* cv Golden orange	fruit	22	2024	0,0071	0,0782	-
CVG 2562	*Colletotrichum acutatum* SC (*C. nymphaeae* clade)	*C. acutatum* SC	*Malus × domestica* cv Golden orange	fruit	22	2024	0,0057	0,0895	-
CVG 2563	*Colletotrichum alienum*	*C. gloeosporioides* SC	*Malus × domestica* cv Story® Inored	fruit	23	2024	0,0544	0,0782	-
CVG 2564	*Colletotrichum gloeosporioides* SC	*C. gloeosporioides* SC	*Malus × domestica* cv Story® Inored	fruit	23	2024	0,0115	0,0967	-
CVG 2565	*Colletotrichum gloeosporioides* SC	*C. gloeosporioides* SC	*Malus × domestica* cv Story® Inored	fruit	23	2024	0,0494	0,0949	-

CVG 3114	*Colletotrichum chrysophilum*	*C. gloeosporioides* SC	*Malus* × *domestica* cv SchniCo Red Gala Schinga®	leaf	24	2025	0,0097	0,0608	0,2915
CVG 3115	*Colletotrichum gloeosporioides SC*	*C. gloeosporioides* SC	*Malus* × *domestica* cv SchniCo Red Gala Schinga®	leaf	24	2025	0,0068	0,0246	0,4824
CVG 3116	*Colletotrichum gloeosporioides SC*	*C. gloeosporioides* SC	*Malus* × *domestica* cv SchniCo Red Gala Schinga®	leaf	24	2025	0,0078	0,0753	0,8148
CVG 3117	*Colletotrichum gloeosporioides SC*	*C. gloeosporioides* SC	*Malus* × *domestica* cv SchniCo Red Gala Schinga®	leaf	24	2025	0,0260	0,0782	-
CVG 3118	*Colletotrichum gloeosporioides SC*	*C. gloeosporioides* SC	*Malus* × *domestica* cv SchniCo Red Gala Schinga®	leaf	24	2025	0,0731	0,0797	0,2625
CVG 3119	*Colletotrichum gloeosporioides SC*	*C. gloeosporioides* SC	*Malus* × *domestica* cv SchniCo Red Gala Schinga®	leaf	24	2025	0,0636	0,0829	0,2625
CVG 3120	*Colletotrichum chrysophilum*	*C. gloeosporioides* SC	*Malus* × *domestica* cv SchniCo Red Gala Schinga®	leaf	24	2025	0,0125	0,0949	0,3232
CVG 3121	*Colletotrichum gloeosporioides SC*	*C. gloeosporioides* SC	*Malus* × *domestica* cv SchniCo Red Gala Schinga®	leaf	24	2025	0,0091	0,0645	0,4285
CVG 3122	*Colletotrichum gloeosporioides SC*	*C. gloeosporioides* SC	*Malus* × *domestica* cv SchniCo Red Gala Schinga®	leaf	24	2025	0,0323	0,0683	-
CVG 3123	*Colletotrichum gloeosporioides SC*	*C. gloeosporioides* SC	*Malus* × *domestica* cv SchniCo Red Gala Schinga®	leaf	24	2025	0,0266	0,0813	0,8469
CVG 3124	*Colletotrichum gloeosporioides SC*	*C. gloeosporioides* SC	*Malus* × *domestica* cv SchniCo Red Gala Schinga®	leaf	24	2025	0,0310	0,0813	0,6462
CVG 3125	*Colletotrichum gloeosporioides SC*	*C. gloeosporioides* SC	*Malus* × *domestica* cv SchniCo Red Gala Schinga®	leaf	24	2025	0,0533	0,0829	0,9511
CVG 3126	*Colletotrichum gloeosporioides SC*	*C. gloeosporioides* SC	*Malus* × *domestica* cv SchniCo Red Gala Schinga®	leaf	24	2025	0,0158	0,0585	0,3878
CVG 3130	*Colletotrichum chrysophilum*	*C. gloeosporioides* SC	*Malus* × *domestica* cv Gala	leaf	5	2025	0,0210	0,0753	0,9511
CVG 3131	*Colletotrichum gloeosporioides SC*	*C. gloeosporioides* SC	*Malus* × *domestica* cv Gala	leaf	5	2025	0,0152	0,0813	-
CVG 3132	*Colletotrichum gloeosporioides SC*	*C. gloeosporioides* SC	*Malus* × *domestica* cv Gala	leaf	5	2025	0,0138	0,0845	0,3957
CVG 3133	*Colletotrichum gloeosporioides SC*	*C. gloeosporioides* SC	*Malus* × *domestica* cv Gala	leaf	5	2025	0,0083	0,0585	0,4037

CVG 3134	*Colletotrichum gloeosporioides SC*	*C. gloeosporioides* SC	*Malus* × *domestica* cv Gala	leaf	5	2025	0,0077	0,2072	-
CVG 3135	*Colletotrichum chrysophilum*	*C. gloeosporioides* SC	*Malus* × *domestica* cv Simmons/Buckeye®	leaf	25	2025	0,0057	0,1297	0,4824
CVG 3136	*Colletotrichum gloeosporioides SC*	*C. gloeosporioides* SC	*Malus* × *domestica* cv Simmons/Buckeye®	leaf	25	2025	0,0055	0,0271	0,7840
CVG 3137	*Colletotrichum nymphaeae*	*C. acutatum* SC	*Malus* × *domestica* cv Simmons/Buckeye®	leaf	25	2025	0,0055	0,0501	-
CVG 3138	*Colletotrichum gloeosporioides SC*	*C. gloeosporioides* SC	*Malus* × *domestica* cv Simmons/Buckeye®	leaf	25	2025	0,0122	0,0463	-
CVG 3139	*Colletotrichum gloeosporioides SC*	*C. gloeosporioides* SC	*Malus* × *domestica* cv Simmons/Buckeye®	leaf	25	2025	0,0113	0,0813	0,3507
CVG 3140	*Colletotrichum gloeosporioides SC*	*C. gloeosporioides* SC	*Malus* × *domestica* cv Simmons/Buckeye®	leaf	25	2025	0,0109	0,0829	0,4458
CVG 3146	*Colletotrichum chrysophilum*	*C. gloeosporioides* SC	*Malus* × *domestica* cv Gala	leaf	26	2025	0,0107	0,0949	-
CVG 3147	*Colletotrichum gloeosporioides SC*	*C. gloeosporioides* SC	*Malus* × *domestica* cv Gala	leaf	26	2025	0,0120	0,0861	0,7398
CVG 3148	*Colletotrichum gloeosporioides SC*	*C. gloeosporioides* SC	*Malus* × *domestica* cv Gala	leaf	26	2025	0,0088	0,0738	-
CVG 3150	*Colletotrichum gloeosporioides SC*	*C. gloeosporioides* SC	*Malus* × *domestica* cv Gala	leaf	26	2025	0,0099	0,0670	-
CVG 3151	*Colletotrichum gloeosporioides SC*	*C. gloeosporioides* SC	*Malus* × *domestica* cv Gala	leaf	26	2025	0,0088	0,0574	0,6216
CVG 3154	*Colletotrichum chrysophilum*	*C. gloeosporioides* SC	*Malus* × *domestica* cv Gala	leaf	5	2025	0,0067	0,0574	0,2463
CVG 3155	*Colletotrichum gloeosporioides SC*	*C. gloeosporioides* SC	*Malus* × *domestica* cv Gala	leaf	5	2025	0,0083	0,1584	0,4201
CVG 3156	*Colletotrichum gloeosporioides SC*	*C. gloeosporioides* SC	*Malus* × *domestica* cv Gala	leaf	5	2025	0,0052	0,1046	0,2570
CVG 3157	*Colletotrichum gloeosporioides SC*	*C. gloeosporioides* SC	*Malus* × *domestica* cv Gala	leaf	5	2025	0,0060	0,0585	-
CVG 3158	*Colletotrichum gloeosporioides SC*	*C. gloeosporioides* SC	*Malus* × *domestica* cv Gala	leaf	5	2025	0,0071	0,0620	-

CVG 3159	*Colletotrichum fioriniae*	*C. acutatum* SC	*Malus × domestica* cv Runsè	fruit	27	2025	0,0026	0,0767	0,0002
CVG 3160	*Colletotrichum acutatum SC* (*C. fioriniae* clade)	*C. acutatum* SC	*Malus × domestica* cv Runsè	fruit	27	2025	0,0053	0,0260	0,0004
CVG 3161	*Colletotrichum acutatum SC* (*C. fioriniae* clade)	*C. acutatum* SC	*Malus × domestica* cv Runsè	fruit	27	2025	0,0068	0,0335	0,0003
CVG 3162	*Colletotrichum godetiae*	*C. acutatum* SC	*Malus × domestica* cv ISAAQ	fruit	28	2025	0,0046	0,0753	0,0002
CVG 3163	*Colletotrichum acutatum* SC (*C. godetiae* clade)	*C. acutatum* SC	*Malus × domestica* cv ISAAQ	fruit	28	2025	0,0042	0,0683	0,0001

Figure S1. Visual reference scale used for the assessment of disease severity on apple leaves in pathogenicity assays. Scores correspond to the following symptomatic leaf-area classes: 0, no symptoms; 1, ≤5%; 2, 6–10%; 3, 11–25%; 4, 26–50%; and 5, 51–100%.

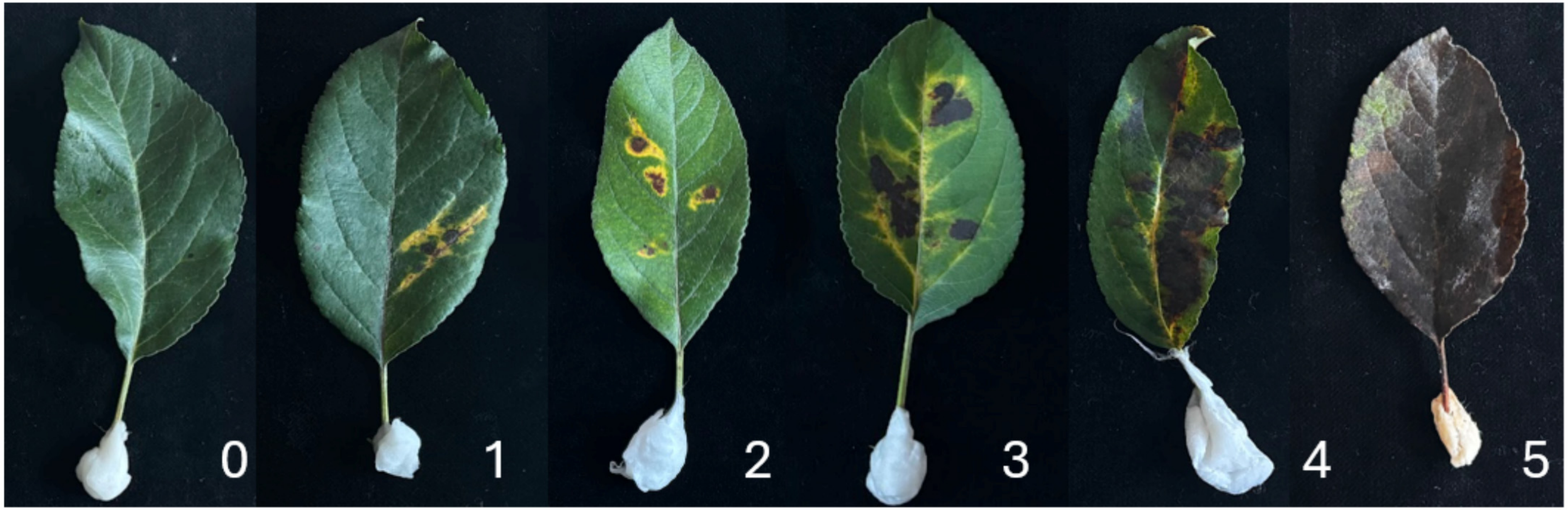

Supplement: Supplementary file 1 [file plants-15-03121-s001.zip › plants-4626144-supplementary.pdf]
